# A tutorial on optical photothermal infrared (O-PTIR) microscopy

**DOI:** 10.1063/5.0219983

**Published:** 2024-09-13

**Authors:** Craig B. Prater, Mustafa Kansiz, Ji-Xin Cheng

**Affiliations:** 1 Photothermal Spectroscopy Corporation, Santa Barbara, California 93111, USA; 2 Photonics Center, Boston University, Boston, Massachusetts 02215, USA

## Abstract

This tutorial reviews the rapidly growing field of optical photothermal infrared (O-PTIR) spectroscopy and chemical imaging. O-PTIR is an infrared super-resolution measurement technique where a shorter wavelength visible probe is used to measure and map infrared (IR) absorption with spatial resolution up to 30× better than conventional techniques such as Fourier transform infrared and direct IR laser imaging systems. This article reviews key limitations of conventional IR instruments, the O-PTIR technology breakthroughs, and their origins that have overcome the prior limitations. This article also discusses recent developments in expanding multi-modal O-PTIR approaches that enable complementary Raman spectroscopy and fluorescence microscopy imaging, including wide-field O-PTIR imaging with fluorescence-based detection of IR absorption. Various practical subjects are covered, including sample preparation techniques, optimal measurement configurations, use of IR tags/labels and techniques for data analysis, and visualization. Key O-PTIR applications are reviewed in many areas, including biological and biomedical sciences, environmental and microplastics research, (bio)pharmaceuticals, materials science, cultural heritage, forensics, photonics, and failure analysis.

## INTRODUCTION

**FIG. 1.**
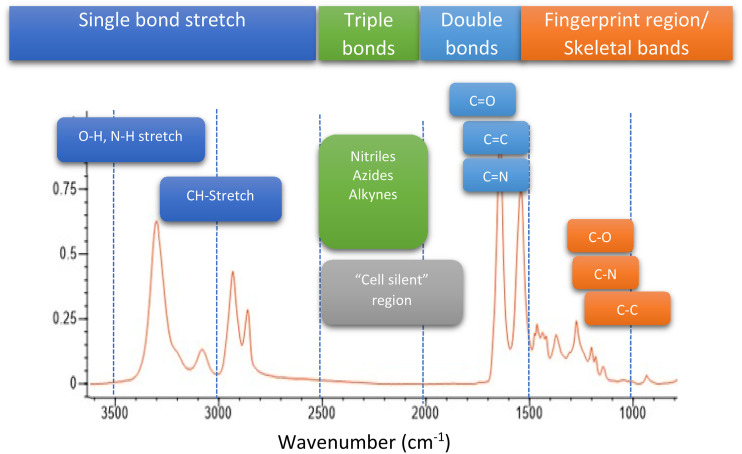
Key functional groups accessible in mid-infrared spectroscopy. The “cell silent” region refers to a region without significant IR absorption by biological materials and is used for stable isotope labeling for metabolic studies.

Infrared spectroscopy is one of the most widely used techniques for chemical analysis with many diverse applications. Mid-infrared wavelengths have oscillation frequencies that correspond to numerous chemical bond vibrations within organic and inorganic molecules (Fig. 1) and thus serve as excellent probes of molecular composition based on chemical functional groups and molecular conformation within a sample. Infrared (IR) spectroscopy enables characterization and identification of materials based on infrared absorption spectra, which can serve as highly specific molecular fingerprints. The combination of infrared spectroscopy with optical microscopy in the 1980s enabled rich spatially resolved chemical analysis. Conventional IR micro-spectroscopy, however, suffers from several key limitations, namely, relatively coarse spatial resolution, complex sample preparation requirements, and issues with scattering/dispersive artifacts. Optical photothermal infrared (O-PTIR) spectroscopy and imaging was developed to address these limitations. This tutorial will review the origins of O-PTIR, the underlying technology, and review recent applications.

### Limitations of conventional infrared spectroscopy

The resolution of a conventional optical microscope, including infrared microscopes, is constrained by optical diffraction. The lateral spatial resolution *d*_*l*_ according to the Rayleigh criterion is given by

$$d_{l}=\frac{0.61\lambda }{NA},$$ (1)

where λ is the wavelength, n is the index of refraction, and NA is the numerical aperture of the focusing and/or collection objective. For mid-IR wavelengths in the range of 2.5–25 *μ*m, this corresponds to a spatial resolution range of around 3–30 *μ*m for commonly used IR objectives. It is important to point out that for traditional infrared spectroscopy, the actual spatial resolution is very much wavelength (wavenumber) dependent, hence there is no singular value that can be used to characterize the spatial resolution performance of an infrared microscope, but rather, there is a range of values, which is typically in the order of 3–30 *μ*m. This spatial resolution is significantly coarser than conventional optical microscopy performed at visible wavelengths. For example, a 0.8 NA air objective imaging at 532 nm can achieve a spatial resolution of around 400 nm, and water or oil objectives can achieve a resolution of ∼250 nm. This has left IR micro-spectroscopy at a significant deficit compared to visible light microscopy. The spatial resolution limit of mid-IR micro-spectroscopy has also constrained its application in critical high-value research areas. For example, in the case of life sciences, the mid-IR spatial resolution limits on the scale of 3–30 *μ*m is similar to the length scale of entire biological cells, making most sub-cellular spectroscopic analysis impossible or extremely limited at mid-IR wavelengths. Somewhat higher spatial resolution can be achieved with attenuated total reflection (ATR), but this approach requires direct mechanical contact of a high index crystal with the sample, which can lead to sample damage, cross-contamination, and inconsistent imaging. Conventional infrared spectroscopy is often preferably performed in a transmission configuration, which requires thin sectioning of samples (to ∼5–10 *μ*m thickness) to avoid excessive absorption by the sample and resulting nonlinearity and saturation effects. Finally, when samples have features on the length scale of mid-IR wavelengths, Mie scattering can cause size, shape, and wavelength-dependent scattering effects that can distort spectra, making spectral interpretation difficult. The O-PTIR approach, as detailed in the following, addresses all these key limitations, providing a non-contact measurement approach with spatial resolution commensurate with visible light optical microscopy, no requirement for thin-sectioning, and that is insensitive to size and shape scattering artifacts.

### The origins of O-PTIR

**FIG. 2.**
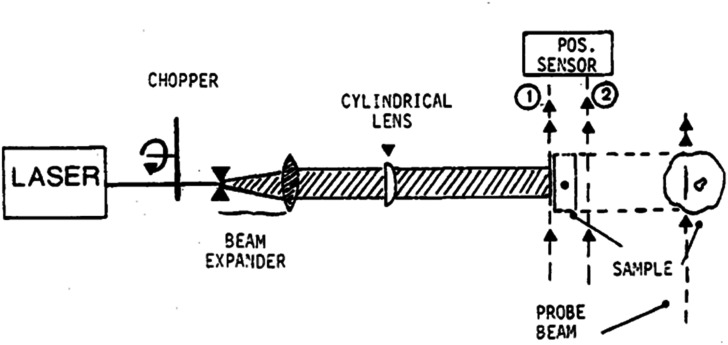
Photothermal imaging apparatus of Fournier *et al.* reproduced with permission from Fournier *et al.*, J. Phys. Colloq. **44**(C6), C6-479 (1983). Copyright 1983 EDP Sciences.

Photothermal infrared spectroscopy has been exploited for decades, primarily through the application of photo-acoustic Fourier transform infrared (PA-FTIR), often also simply referred to as photo-acoustic spectroscopy (PAS). PA-FTIR was based on the absorption of IR light into a solid sample, with the signal detected by the subtle acoustic waves generated by sample expansion, typically in a nitrogen atmosphere with an ultra-sensitive microphone.^1^ While being a very useful sampling accessory to FTIR, especially for the more bulk, intractable samples, it lacked any spatial resolving capabilities. Photothermal imaging has a history dating back at least to the 1980s, including the work of Fournier *et al.*^2^ (Fig. 2) who used the “mirage effect” to detect absorption of laser radiation by a sample. Despite a rich application space for photothermal imaging and spectroscopy that emerged since this work, almost thirty years passed before it was realized that photothermal detection approaches could be used to overcome infrared diffraction limits that constrain mid-IR spectroscopy.

The first approaches for overcoming the mid-IR diffraction limit originated in the field of atomic force microscopy where the probe of an atomic force microscope was used to detect thermal expansion resulting from absorption of IR light.^3–6^ The AFM-IR technique has become widely used, but its applications have been constrained by the level of technical skill required to operate the AFM, as well as limits on measurement speed and samples that can be readily measured by AFM. In 2012, researchers at the U.S. Naval Research Laboratory^7^ reported the use of an optical probe beam operating at visible wavelengths to detect IR absorption at a spatial resolution smaller than the diffraction limit for mid-IR light. Researchers at Boston University reported a similar work by using a quantum cascade laser (QCL) as the excitation source.^8,9^ Yet, these preliminary research described in conference proceedings did not demonstrate super-resolution over IR microscopy, nor the potential of imaging a living system by an infrared pump and a visible probe.

In 2011, Cheng, a co-inventor of coherent Raman scattering microscopy,^10^ independently started to explore infrared photothermal microscopy at Purdue University to overcome the sensitivity issue in Raman microscopy due to the very low cross section of Raman scattering. After five years of persistent effort in 2016, the Cheng group reported use of dark-field beam geometry and a resonant circuit to pick up the photothermal signal, to enable a high-performance mid-infrared photothermal (MIP) microscope that allowed for the first time 3D imaging of chemical bonds inside a living cell at 600 nm resolution and 10 *μ*M limit of detection.^11^ The pioneering studies at Purdue^11–13^ and independent work at Notre Dame^14–16^ quickly inspired new developments by various groups (for reviews^17,18^) and caught industrial attention. The first commercial MIP instrument “mIRage” was announced in October 2017 by Anasys Instruments who had previously commercialized AFM-based photothermal infrared spectroscopy. The acronym O-PTIR (optical photothermal infrared) was coined to differentiate the technology from the AFM-based photothermal infrared approach. For avoidance of confusion, O-PTIR and MIP refer to the same super-resolution IR spectroscopy and chemical imaging approach. The acronym O-PTIR will be used for the remainder of this review. Photothermal Spectroscopy Corp. (a spinoff of Anasys) was founded in 2018 to develop and commercialize this emerging technology with full momentum, in collaboration with the Cheng group, which moved to Boston University in 2017. Two prior review articles^17,18^ provide an excellent summary of academic research in the field of super-resolution infrared photothermal microscopy. In this tutorial, we introduce the working principle, instrumentation, and applications of OPTIR microscopy in lay language.

### How O-PTIR works

**FIG. 3.**
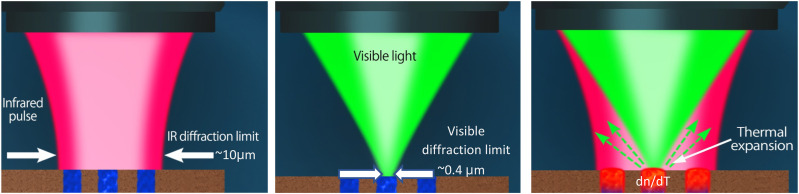
O-PTIR uses a visible probe beam to detect infrared absorption with sub-500 nm spatial resolution via photothermal detection sensitive to thermal expansion and index of refraction changes in IR absorbing regions of a sample.

The O-PTIR approach (Fig. 3) works by illuminating a sample with pulses of IR radiation, typically from a tunable IR laser source, such as a quantum cascade laser (QCL) and detecting localized heating of IR absorbing regions with a shorter wavelength visible probe beam. In particular, when IR light excites molecular bonds within a sample, a portion of the energy absorbed and dissipated in the sample, resulting in localized heating. The resulting temperature increase causes thermal expansion in the sample that, in turn, causes a reduction of the index of refraction of the IR-heated regions. The optical probe beam can detect these IR-induced temperature changes because the thermal expansion and associated reduction in the index of refraction alter the intensity and angular distribution of probe light reflected, scattered, and/or transmitted by the sample.

### Achieving super-resolution photothermal infrared

**FIG. 4.**
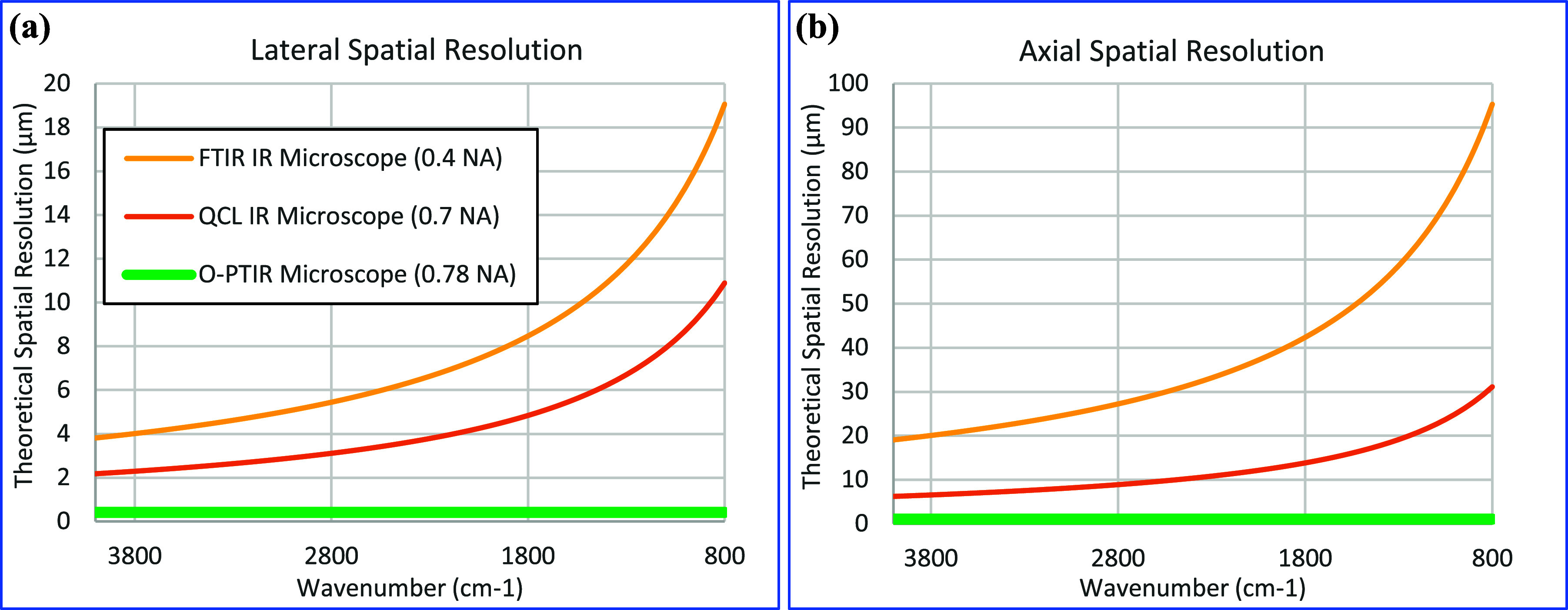
Comparison of theoretical lateral (a) and axial (b) spatial resolution of conventional IR microscopes (FTIR and QCL based) with the O-PTIR approach. Unlike conventional IR microscopes, O-PTIR’s spatial resolution is set by the probe wavelength and thus is constant over all IR wavelengths.

The O-PTIR approach achieves spatial resolution commensurate with the visible light optical microscope and Raman microscope because the spatial resolution is set by the optical diffraction limit of the shorter wavelength probe beam, not the longer wavelength IR beam. Because the probe beam wavelength is chosen to be roughly an order of magnitude shorter than mid-IR wavelengths, the achievable resolution achievable by O-PTIR [as indicated by Eq. (1)] is roughly an order of magnitude better than conventional IR instrument. Figure 4(a) shows a comparison of the theoretical lateral spatial resolution of typical conventional IR microscopes (both FT-IR and QCL based). For example, at 1000 cm^−1^ (10 *μ*m IR wavelength), a typical FT-IR based microscope with a 15×, 0.4 NA objective would achieve a lateral spatial resolution of around 15 *μ*m, whereas an O-PTIR instrument operating with a 532 nm probe wavelength and a 0.78 NA objective would achieve a theoretical spatial resolution of 416 nm, an improvement of ∼36×. The O-PTIR approach thus enables “super-resolution” infrared microscopy/spectroscopy by exceeding the optical diffraction limit for infrared wavelengths. For the avoidance of confusion, the term “super-resolution” in this context does not refer to approaches used in optical microscopy to exceed the ∼250 nm diffraction limit of visible light microscopy, but rather exceeding the corresponding diffraction limits for infrared wavelengths. O-PTIR also achieves significant improvements in axial resolution, as shown in Fig. 4(b). Axial resolution *d*_*a*_, as given by Eq. (2) depends on the square of the NA, causing lower NA objectives commonly used in FT-IR microscopy to suffer very coarse axial spatial resolution e.g., around 76 *μ*m at 1000 cm^−1^ with a 0.4 NA objective, whereas a typical O-PTIR microscope can achieve an axial spatial resolution around 1 *μ*m at the same wavelength,

$$d_{a}=\frac{2\lambda }{NA^{2}}.$$ (2)

Even higher lateral and axial resolutions can be achieved in O-PTIR using higher NA refractive objectives, which are unsuitable for FTIR/QCL microscopy and using immersion objectives and/or confocal microscopy approaches, as will be discussed later.

### O-PTIR instrumentation

**FIG. 5.**
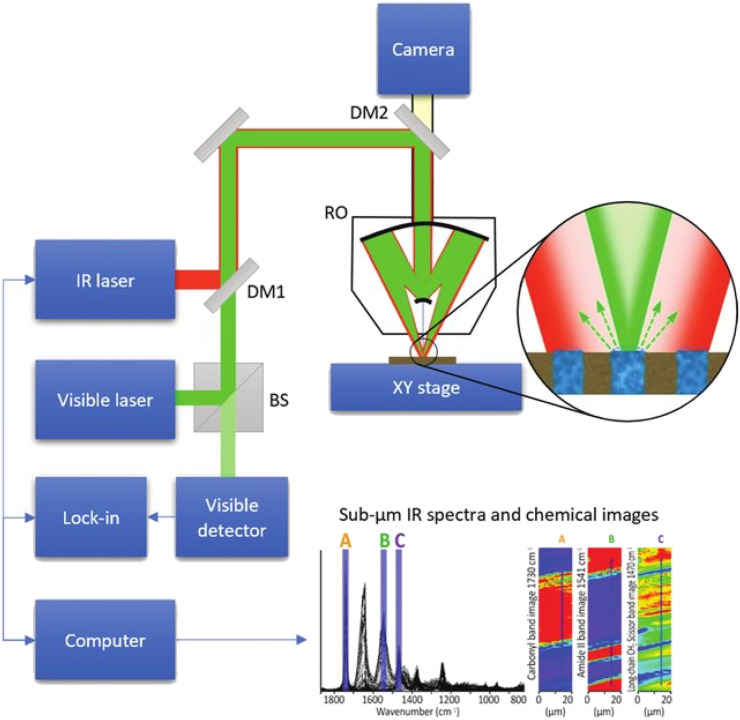
Simplified schematic diagram of an O-PTIR instrument. Reprinted with permission from Kansiz *et al.*,^24^ Microsc. Today **28**(3), 26 (2020). Copyright 2020 Microscopy Society of America.

An example schematic of O-PTIR instrumentation is shown in Fig. 5. In this arrangement, infrared light from a tunable IR laser is combined coaxially with light from a visible probe laser using a dichroic mirror DM1, and both IR and probe beams are focused onto the sample using a reflective objective (RO) of a Schwarzschild design (also called Cassegrain objective). A reflective objective is necessary due to the ultra-wide range of wavelengths being employed, from visible wavelength (400–700 nm) to IR wavelengths up to about 12 *μ*m. In addition, typical glass objectives used in optical or Raman microscopy are not transmissive to mid-IR wavelengths. Absorption of IR light by the sample creates a modulated local temperature increase that causes both thermal expansion of the sample and a resulting decrease in the local index of refraction due to the decrease in sample density. The changes in size, shape, and index of refraction alter the intensity and angular distribution of light reflected, scattered, and/or transmitted by the sample. The typical magnitude of fractional change in the index of refraction is around 10^−4^/°C. The IR source is typically pulsed at frequencies in the range of 100 kHz to produce periodic modulations in the intensity/distribution of the probe beam. In the configuration shown, modulated probe light is collected by the same reflective objective and sent to a photodetector. In other configurations discussed later, the modulated probe beam can also be collected in the transmission directions. The detector signal is generally demodulated with a lock-in amplifier^11^ at the pulse repetition frequency of the IR laser or a harmonic thereof.^19–21^ Demodulation in the time domain has also been demonstrated,^22,23^ which can provide time-resolved analysis of the samples’ photothermal response. Modulation of the probe beam is then measured as a function of IR wavelength (wavenumber) to create IR absorption spectra or as a function of the sample position to create IR absorption images at desired IR absorption bands.

**FIG. 6.**
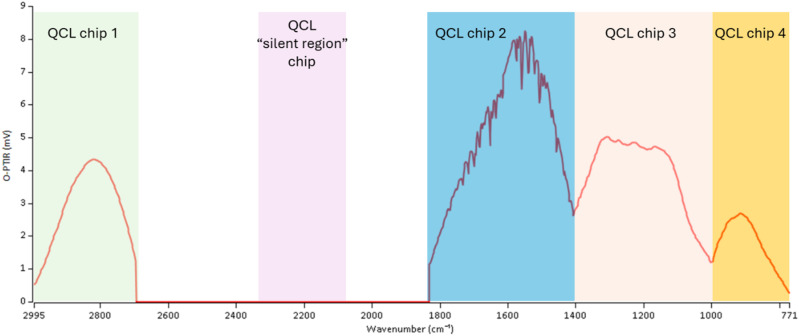
Example QCL power curve for a four chip QCL covering the CH region (QCL chip 1) and fingerprint region (QCL chips 2–4). QCLs are also available with emission in the “silent region” for metabolic studies, as discussed later.

Current generation QCLs are typically composed of four “chip” slots, which are user-configurable with an example power spectrum, as shown in Fig. 6, of a common, so-called “C–H/Fingerprint” QCL. More recently, interest in silent region IR tags (discussed further below) has increased; one could configure a four chip, as a so-called tri-range QCL covering the C–H region, silent region, and fingerprint region out to the high 900 cm^−1^. Collected IR spectra are normalized for the variable, wavelength dependent power output that is typical of QCL as well as optical path and atmospheric (e.g., water vapor) absorbances (e.g., the downward spikes in the QCL chip 2 power curve in the following).

## KEY O-PTIR CAPABILITIES

Among the key advantages of O-PTIR are the ability to achieve sub-500 nm spatial resolution at mid-IR wavelengths, the ability to acquire spectra with excellent correlation to conventional FT-IR transmission spectra, and high sensitivity/signal-to-noise ratio. Each of these are discussed briefly in the following.

### Sub-500 nm super-resolution IR imaging

**FIG. 7.**
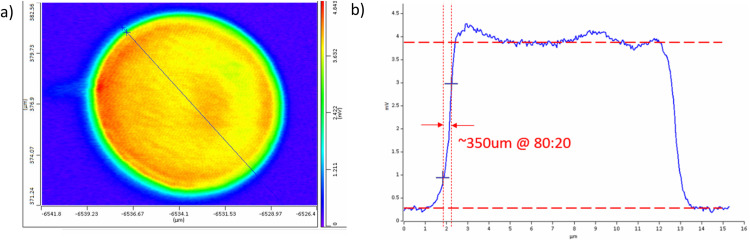
Super-resolution infrared chemical imaging with O-PTIR. The sample is an (a) IR chemical image of PMMA bead at 1730 cm^−1^ with 50 nm pixel size. (b) Cross section of IR absorption across the line indicated in panel (a) showing spatial resolution at the PMMA/epoxy boundary of around 350 nm.

Figure 7 in the following shows an example IR absorption image demonstrating sub-500 nm spatial resolution. For this test, a 10 *μ*m diameter PMMA bead was embedded in epoxy and sectioned to 500 nm thick and then imaged in counter-propagating mode (described in a following section) at 1730 cm^−1^, which is strongly absorbed by the carbonyl band in PMMA. A cross section of the IR absorption shown in Fig. 7(b) reveals a spatial resolution of around 350 nm. A similar resolution has been reported in the literature on 100 nm polystyrene beads,^16^ lipid droplets in cells,^25^ and other samples. Even higher spatial resolution has been demonstrated using higher-harmonic detection approaches.^20^

### Rapid high-quality spectra

IR absorption spectra are acquired by using O-PTIR by measuring the modulation of the collected visible probe beam while sweeping the wavelength of the tunable IR source. The acquisition speed is determined by the sweep speed of the tunable IR laser source and the desired signal to noise ratio (SNR). The most commonly used IR laser sources are quantum cascade lasers (QCLs), which can be tuned at rates of 1000 cm^−1^/s and higher. IR spectra are typically acquired in 1–5 s depending on the spectral resolution and SNR requirements for the measurement. For higher SNR, multiple spectra can be acquired and co-averaged. In typical operation, a user will acquire some exemplar spectra at various locations on the sample to determine the IR absorption bands present in the sample. Examination of the collected spectra can reveal which IR bands show the most variation between different constituents in the sample. With this knowledge, it is possible to acquire IR absorption images at multiple IR absorption bands to reveal the spatial heterogeneity within the sample. It is also possible to collect hyperspectral arrays of O-PTIR spectra over an array of locations on a sample, or using emerging wide-field approaches acquire an array of IR absorption images at different IR wavelengths. These hyperspectral arrays can provide a rich analysis of the spatial and spectral variations in complex samples.

### Strong correlation to FTIR spectra

**FIG. 8.**
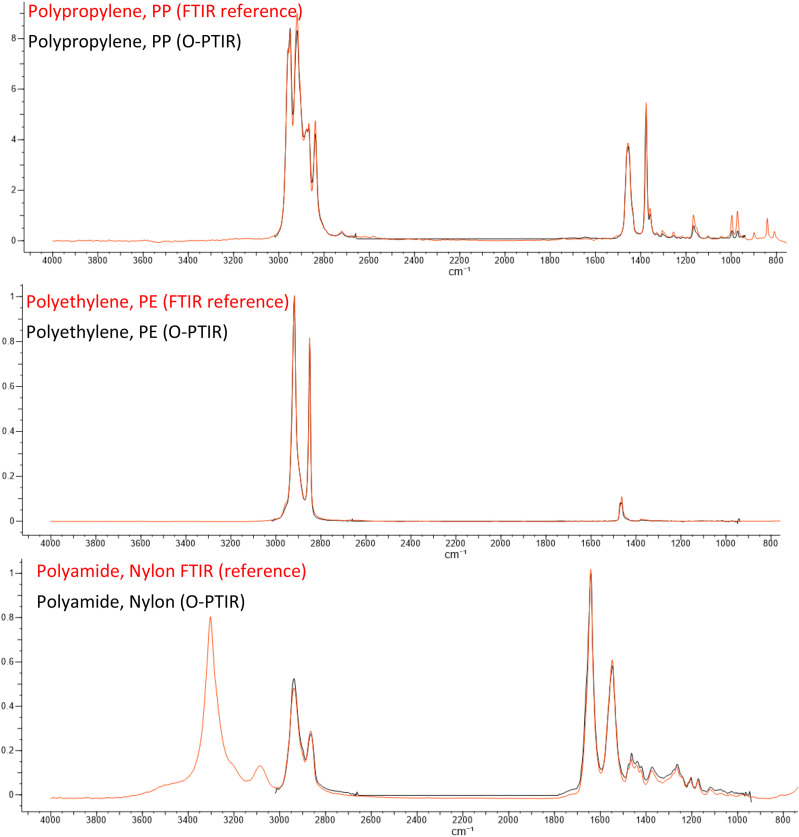
Comparison of O-PTIR spectra with FT-IR reference spectra. (Reference spectra source: Wiley Know-It-All).

One of the strengths of the O-PTIR approach is the ability to acquire super-resolution infrared spectra with very strong correlation to transmission-mode FT-IR spectra. Figure 8 shows an example O-PTIR spectra measured on a variety of polymeric films in comparison with conventional transmission-mode FT-IR spectra from a material reference database. The reason for the strong correlation is that the signal detected in O-PTIR, a modulation in the collected probe beam, is directly proportional to the spectral absorbance that is measured in FT-IR instruments. This has enabled researchers to leverage large spectral databases with hundreds of thousands of IR spectra for the identification of unknowns. This correlation has proven very useful, for example, in the analysis/identifications of organic contaminants,^24^ nanocomposites,^26^ and microplastic particles.^27^

The reason for this correlation is that in most cases: (1) the temperature increase in the sample varies linearly with the sample IR absorption coefficient and (2) O-PTIR signal is generally linear with the increase in sample temperature. This is summarized in the following in a simplified analysis of relevant photothermal physics, following an analogous analysis for AFM-IR by Dazzi and Prater (see supplementary material of Ref. 6). First, as shown in Eq. (3), the IR spectral absorbance $A\left(\nu \right)$ as a function of wavenumber *ν* is proportional to the wavenumber and the sample’s imaginary index of refraction *κ*(*ν*),

$$A\left(\nu \right)=\log\left(\frac{1}{T}\right)=\frac{4\pi }{\ln\left(10\right)}\nu \kappa \left(\nu \right).$$ (3)

The amount of heat absorbed by the sample is similarly proportional to the input power *I*_0_, the spectral absorbance $A\left(\nu \right)$, the sample volume *V*, and various constants subsumed in *α*_*opt*_,

$$P_{\mathrm{abs}}\left(\nu \right)=I_{0}\alpha_{\mathrm{opt}}VA\left(\nu \right).$$ (4)

The maximum sample temperature increase is, in turn, proportional to the absorbed IR power, the IR pulse duration *t*_*p*_, and inversely related sample’s density and heat capacity *ρc*_*p*_,

$$\Delta T_{\max}=\frac{P_{\mathrm{abs}}\left(\nu \right)t_{p}}{V\rho c_{p}}=\frac{I_{0}\alpha_{\mathrm{opt}}t_{p}}{\rho c_{p}}A\left(\nu \right).$$ (5)

The increase in sample temperature results in a change in the index of refraction of the sample that, in turn, alters the intensity and distribution of the probe light collected by the detector. The math associated with the intensity change is beyond the scope of this manuscript and dependent on the specific collection geometry, but various models^28–33^ exist within the photothermal physics community. However, the literature and experiments show that, in general, the collected probe intensity will have a linear dependence on sample temperature change in the limit of fractionally small modulation, i.e., $\frac{\Delta {\text{I}}_{\text{PT}}}{I_{dc}}\ll 1$, where *ΔI*_*PT*_ is the fluctuation of the collected probe beam in response to IR absorption and *I*_*dc*_ is the dc intensity at the detector. The collected probe beam intensity modulation can have a complex nonlinear dependence on the sample thermal expansion and temperature induced index of refraction shift, but this dependence can generally be linearized due to the small fractional changes involved. Both the thermal expansion coefficient and the temperature dependence of the index of refraction have a typical fractional sensitivity on the order of 10^−4^/°C for most biological and polymeric materials. This leads to a case where the sample thermal expansion $\frac{\delta h}{h}\ll 1$ and the index change $\frac{\delta n}{n}\ll 1$, providing an essentially linear dependence of collected probe light modulation with temperature. In practical experiments, $\frac{\Delta {\text{I}}_{\text{PT}}}{I_{dc}}$ typically has a maximum value of order 10^−3^ with peak temperature increases on the scale of 1–10 °C. Considering just the index of refraction effects for simplicity, the detected change in the collected probe beam intensity *ΔI*_*PT*_ is linearly related to the spectral absorbance $A\left(\nu \right)$, as shown in Eq. (6). Similar arguments apply to photothermal contrast associated with shape change/surface motion associated with thermal expansion,

$$\Delta {\text{I}}_{\text{PT}}\propto \frac{\delta n}{dT}\Delta T_{\max}=\left(\frac{I_{0}\alpha_{\mathrm{opt}}t_{p}}{\rho c_{p}}\right)\frac{\delta n}{dT}A\left(\nu \right).$$ (6)

For this reason, it is important in O-PTIR to apply ratiometric approaches to normalize for these other signal contributions.

### O-PTIR + Raman

**FIG. 9.**
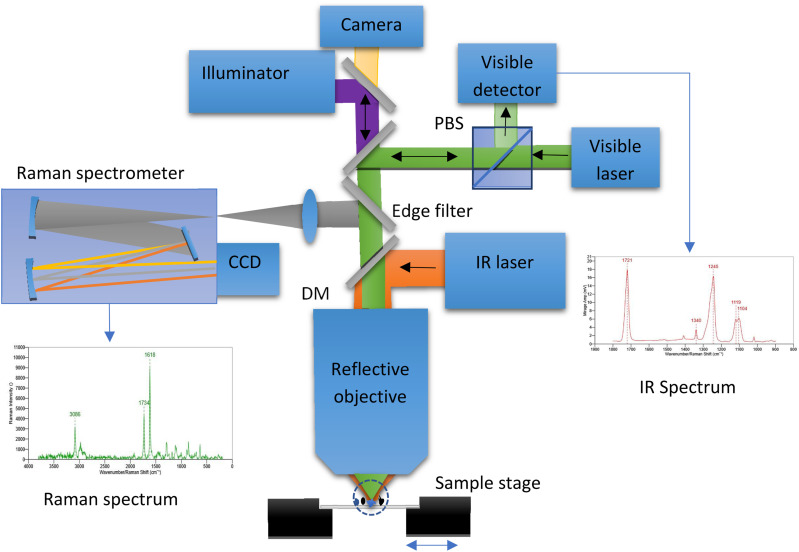
Schematic diagram of instrumentation for combined O-PTIR + Raman spectroscopy.

O-PTIR also supports simultaneous infrared and Raman spectroscopy, leveraging the probe beam for two complementary spectroscopic measurements (Fig. 9). Raman and infrared spectroscopy, while both being vibrational spectroscopic techniques and thus probing molecular vibrations, have very different selection rules (conditions that result in a vibrational signal). For infrared spectroscopy, IR active bands require an inter-atomic vibration that results in a net change in the bond dipole moment. Thus, IR spectroscopy, in general, is more sensitive to bonds involving more polar atoms. On the other hand, Raman spectroscopy selection rules stipulate that for a vibration to be Raman active, the vibration in question must have a change in polarizability during the vibration. Thus, and in contrast to IR spectroscopy, Raman spectroscopy is generally more sensitive to non-polar vibrations. Hence, the two techniques are often considered “complementary” to one another, with molecules with strong IR absorbance, typically having a weaker Raman response and molecules with a strong Raman response, typically having weakening IR absorbances. While the beneficial nature of combining these two complementary techniques for a more thorough spectroscopic characterization is clear, the very different instrument operation, measurement sample presentations required, have meant that true complementary measurements have not been possible, since two very different instrument platforms, with very different achievable spatial resolutions have existed so far.

Therefore, one of the biggest vibrational spectroscopic value propositions of O-PTIR has been that, for the first time, IR and Raman spectroscopy can be collected from the same spot, at the same time, and at the same spatial resolution, thus finally achieving full complementarity of these two techniques.^21,24,34–38^ This can be achieved by using a visible probe beam used to detect IR absorption that is also a low noise narrow bandwidth laser and can thus act and double as a Raman excitation laser. For example, commercial O-PTIR instruments are currently available with 532 and 785 nm laser lines. After interacting the probe beam with the sample, any Raman scattered light is separated by a dichroic mirror (a Raman edge filter) and the light at the original excitation wavelength is sent to a visible room temperature detector for demodulation of the IR absorption signal, whereas wavelength shifted light is sent to a Raman spectrometer for the generation of Raman spectra. As such, the IR and Raman measurements are obtained at the same time, same location, and same spatial resolution, with essentially no compromise on either IR or Raman spectral quality. In this approach, the full complementarity and confirmatory nature of these two techniques is realized, without the need to move the sample, the objective, or any other optic, hence also providing for exact registration between these two channels.

**FIG. 10.**
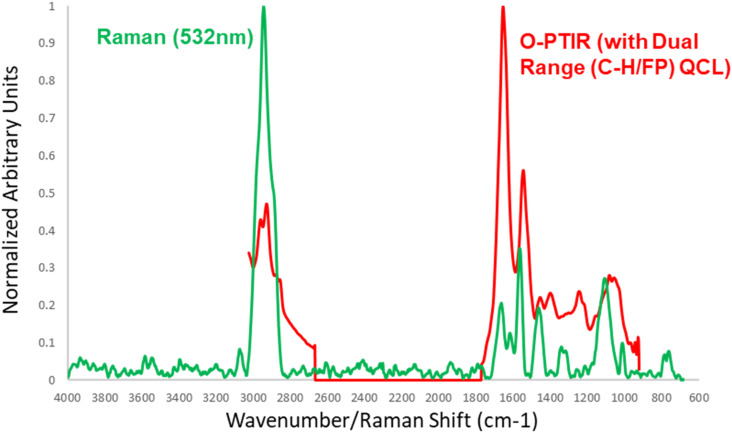
Example of simultaneous and co-located infrared (O-PTIR) and Raman spectra acquired on a single *E. coli* bacterium. The sample is courtesy of Goodacre. Figure courtesy of Photothermal Spectroscopy Corp., reprinted with permission.

An example of a simultaneous, submicron IR + Raman measurement is shown in the following in Fig. 10 of a single *E. coli* bacterium. This measurement is also a good demonstration of the spectroscopic complementarity between IR and Raman. Figure 10 shows very strong Raman C–H bands, which are a characteristic of Raman, while fingerprint spectral features are weaker in Raman. This is generally and conversely true in the IR, as clearly demonstrated in this example with very strong protein (amide I and amide II) bands together with an overall significantly better SNR for the IR (O-PTIR) measurement, despite measurement time being identical at a few seconds of averaging.

### O-PTIR + fluorescence

Fluorescence microscopy has also been combined with O-PTIR to provide labeled targeting of infrared analysis and enhanced measurement sensitivity.

#### Fluorescence-guided O-PTIR

**FIG. 11.**
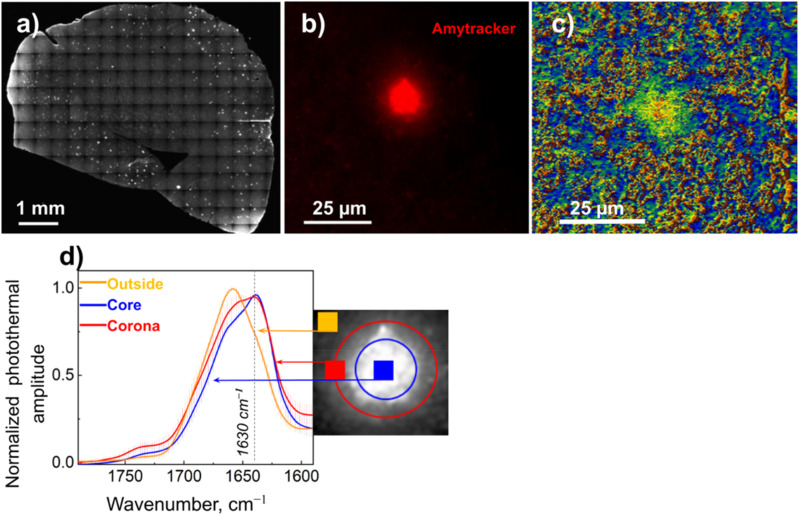
Fluorescence-guided O-PTIR of amyloid plaques in brain tissue. (a) Multi-image fluorescence microscope mosaic of mouse brain tissue with amyloid plaques labeled with Amytracker 520. (b) Zoomed image of an amyloid plaque stained with Amytracker. (c) A map demonstrating the distribution of β-sheet structures as a ratio of the bands at 1630–1656 cm^−1^ for the plaque shown in panel (b). (d) Averaged and normalized O-PTIR spectra were recorded from the outside, core, and plaque corona; the spectra locations were indicated by the markers of the corresponding color on the inset. Reprinted with permission from Prater *et al.*, J. Med. Chem. **66**(4), 2542 (2023). Copyright 2023 Author(s), licensed under a Creative Commons Attribution 4.0 License.

Fluorescence labeling, e.g., by immunofluorescence staining or fluorescent proteins, is ubiquitous in the life sciences and is frequently used to label specific substructures within cells and tissue. Fluorescence imaging while providing excellent chemical specificity by virtue of targeted tabs/labels lacks any broad chemical characterization capabilities. These fluorescent labels can then be used as landmarks to guide targeted chemical analysis via O-PTIR.^39,40^ Although fluorescence labeling has very high specificity, immunofluorescence labeling adheres fluorescent dyes to specific epitopes, but the fluorescence labeling and imaging does not provide information about the specific chemical molecular structure/conformation of the labeled molecules. O-PTIR provides highly complementary analysis capabilities by probing the molecular structure and conformation, which can provide key insights into, for example, the local protein folding state. The group of Prater *et al*. in collaboration with researchers at Photothermal Spectroscopy Corp. demonstrated the ability to use immunofluorescent labeling of amyloid plaques in brain tissue to locate the whereabouts of these amyloid plaques, thus allowing a direct targeting of the O-PTIR analysis of labeled amyloid plaques and adjacent normal tissue to provide for a broad chemical analysis, including protein secondary structure analysis, both of which would be impossible with fluorescence imaging alone.^39^ This analysis demonstrated clear spatially resolved differences in the presence of beta sheet structures associated with protein misfolding, as shown in Fig. 11.

#### Wide field fluorescence detected O-PTIR (FL-PTIR)

Fluorescence emission can also provide an enhanced method of detecting IR absorption. As mentioned previously, conventional O-PTIR detects IR absorption by measuring a modulation in the collected probe beam due to thermal expansion and index of refraction changes with a typical intrinsic photothermal sensitivity of most materials of around 10^−4^/°C (i.e., the change in the index of refraction of a sample with temperature). The efficiency of fluorescence emission (quantum yield), however, is highly temperature dependent with a typical temperature coefficient of around 1%/°C. Researchers at Photothermal Spectroscopy Corp.,^41^ Boston University,^42^ and Purdue University^43,44^ realized that the high temperature of fluorescence emission could provide a new mechanism of detecting local IR absorption with roughly 100× better sensitivity than conventional O-PTIR. The group of Cheng at Boston University demonstrated high sensitivity chemical imaging of fluorescently labeled bacteria and live cancer cells, as well as the ability to perform high-speed wide-field O-PTIR taking advantage of the ∼100× sensitivity enhancement provided by fluorescence detection.^42^ The group of Garth Simpson demonstrated sensitive characterization of the chemical composition within phase-separated domains of amorphous solid dispersions, highlighting its utility in pharmaceutical material analysis^43^ and the ability to use two-photon autofluorescence to enabled fluorescence detected O-PTIR on unlabeled samples.^44^ Another significant benefit to fluorescence detected photothermal infrared (FL-PTIR) is that the fluorescence detector typically employed a high sensitivity wide-field fluorescence camera, and thus operated analogously to infrared focal plane arrays commonly used in FT-IR imaging, i.e., permitting simultaneous measurements of IR absorption at hundreds of thousands of pixels simultaneously. Such operation provides for true wide-field snapshot O-PTIR where all pixels in the array (typically 512 × 512), even up to 5 frames/s with a typical projected pixel size of 130 nm, thus covering a field of view (FOV) of 66 × 66 *μ*m^2^ per tile. This rapid, multi-channel collection process has also provided a massive increase in hyperspectral measurements, with typical full spectral range hyperspectral measurements taking only tens of minutes and with full FOV single frequency images taking seconds (or less). Since the IR source is generally defocused for wide-field measurements, the field of view is generally limited by the power density of the IR source (a QCL in this case). As more powerful IR sources become available, addressable fields of view will also increase. In the meantime, large fields of view are achievable by tiling multiple adjacent measurements in a mosaic.

Recently, a collaboration between Photothermal Spectroscopy Corp. and the group of Kathleen Gough at the University of Manitoba demonstrated high-speed wide-field O-PTIR of biological samples using intrinsic autofluorescent emission instead of fluorescent labeling.^45^ Autofluorescent emission is very common among biological materials, for example, associated with aromatic amino acids, cross-linking, and photosynthetic molecules. Furthermore, autofluorescence is a well-known and much maligned interferent in Raman spectroscopy but can also be an interferent in fluorescence imaging too. In fact, the more autofluorescent a sample, the more sensitive the FL-PTIR measurement will be. The recent work with Kathleen Gough demonstrated the ability to acquire IR hyperspectral arrays on biological materials with high spectral resolution (4 cm^−1^) and sub-500 nm spatial resolution within minutes, including measurements on diatoms, plant tissue, and even dynamic measurement on live microalgae.^45^

## O-PTIR CONFIGURATIONS/MEASUREMENT MODALITIES

**FIG. 12.**
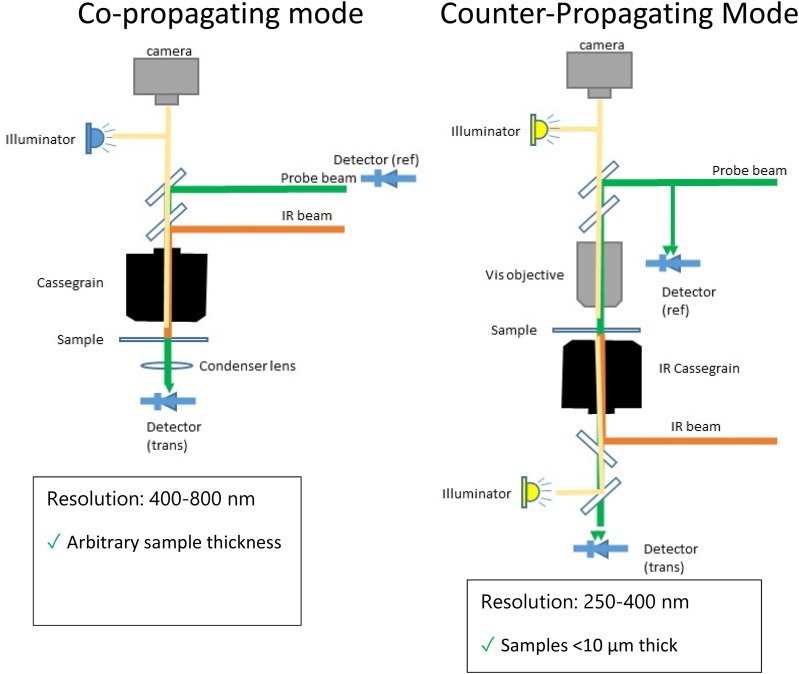
Comparison of co-propagating and counter-propagating O-PTIR measurement modes. Co-propagating mode can be used for any sample thickness with slightly coarser spatial resolution. Counter-propagating mode can achieve higher spatial resolution but requires thinner samples (≲10 *μ*m thick).

**FIG. 13.**
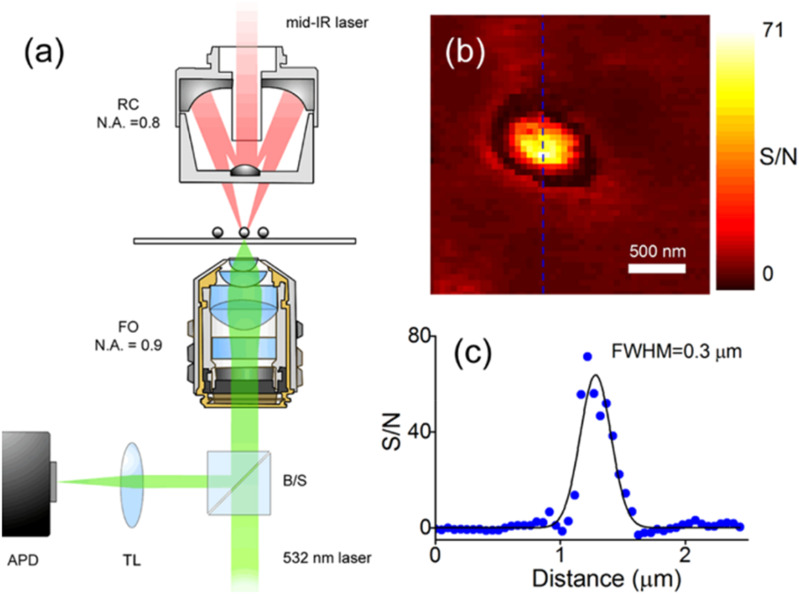
Counter-propagating geometry developed by the researchers at Notre Dame achieving spatial resolution of around 300 nm. (a) Schematic diagram of the apparatus showing the mid-IR pump and 532 nm probe beams focused on the sample with separate objectives. The change in reflectivity of the probe is monitored by an APD with a lock-in amplifier. RC = reflective Cassegrain, FO = focusing objective, B/S = beam splitter, and TL = tube lens. (b) O-PTIR image of a 0.1 *µ*m diameter polystyrene bead recorded with a step size of 0.05 *µ*m. (c) Line profile extracted from the image in panel (b) showing a full width at half maximum (FWHM) of 0.3 *µ*m. Reprinted with permission from Li *et al*., J. Phys. Chem. B **121**(37), 8838 (2017). Copyright 2017 Author(s), licensed under ACS Open Access License.

**FIG. 14.**
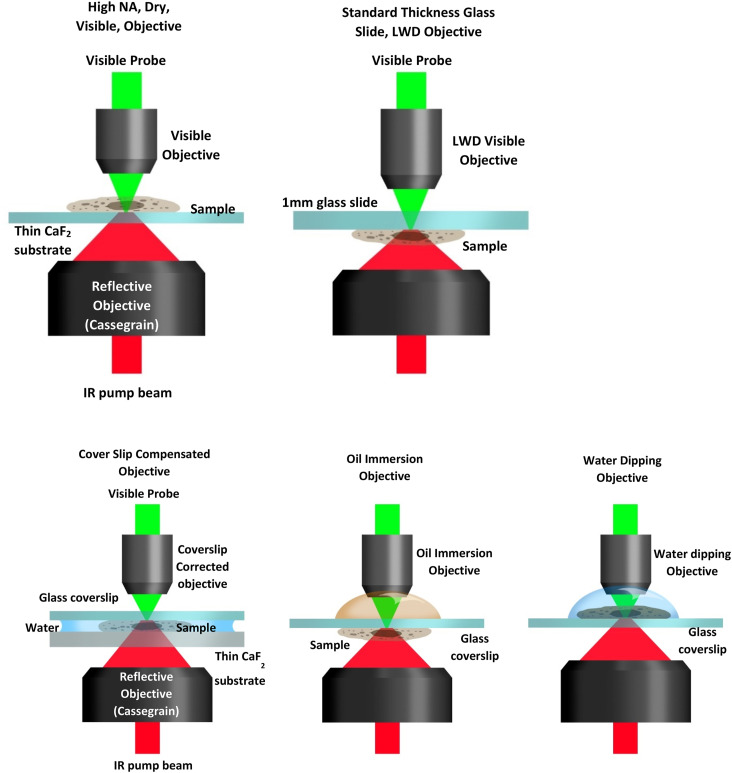
Various objective/sample configurations for measurement in counter-propagating mode.

O-PTIR is usually operated in one of two optical configurations, co-propagating and counter-propagating (Fig. 12). In co-propagating mode, the IR and visible probe beams are arranged to be collinear and illuminate the sample from the same side, top-down. In counter-propagating mode (first demonstrated by the Hartland and Kuno groups at Notre Dame;^14–16^ Fig. 13), the IR beam and probe beam are de-coupled and delivered by separate objectives on opposite sides of the sample. In counter-propagating mode, the IR beam is typically delivered via a reflective objective (from beneath), whereas the probe beam is delivered by a high-quality, high-NA refractive objective (from the top). The counter-propagating approach can achieve higher spatial resolution because refractive objectives can generally attain higher numerical aperture and achieve better optical performance than reflective (Cassegrain) objectives. Furthermore, refractive objectives not only deliver better optical throughput due to their higher collection efficiencies, especially in high NA objectives, but also benefit from the absence of a central obscuration. Central obscuration is an unavoidable drawback in reflective objectives and typically blocks 25%-50% of the incident light. For example, a typical maximum NA for a reflective IR objective is around 0.78, whereas optical objectives are readily available at 0.95 NA for operation in air or up to around 1.2 for water immersion objectives, which deliver >50% improvement in spatial resolution relative to co-propagation operation with reflective Cassegrain objectives. (Oil immersion objectives with NA up to 1.4 can provide even better spatial resolution.) Thus counter-propagating mode can achieve a best case spatial resolution of around 200–250 nm, whereas co-propagating mode achieves spatial resolution in the 400–800 nm range depending on the specific objective NA and probe wavelength. However, counter-propagation mode typically requires the use of thin IR transparent substrates (often 350 *μ*m thick CaF_2_ windows) and requires thin samples, i.e., not thicker than ∼5–10 *μ*m to avoid excessive self-absorption and/or scatter of the incident IR light. The use of counter-propagating mode on thicker samples or highly scattering samples can lead to distortions in the spectra resulting from attenuation/scattering of the infrared beam before it interacts with the surface where the probe beam is focused. A key advantage of co-propagating mode is that it can measure samples of arbitrary thickness and on opaque substrates, at the expense of somewhat worse spatial resolution. Thus, it is often beneficial to have access to both modes of operation with switching between these modes typically achieved through instrument software selection. Examples of possible counter-propagating objectives, sample presentation (upright vs upside-down), and substrate choice configurations combinations are shown in Fig. 14.

**FIG. 15.**
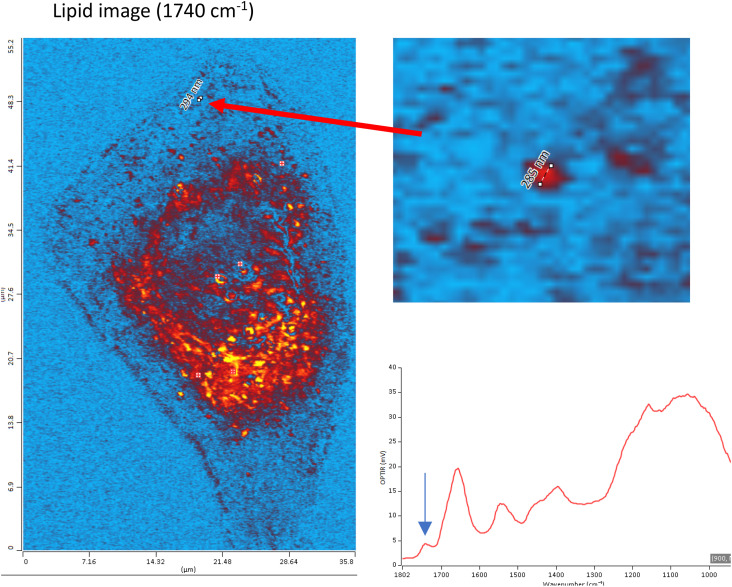
Sub-300 nm spatial resolution O-PTIR image acquired in counter-propagating mode with a 1.3 NA oil objective on a dried biological cell at the 1740 cm^−1^ absorption peak indicated with the blue arrow on the spectrum at the bottom right. The sample is courtesy of Gough, measurement by Photothermal Spectroscopy Corp., used with permission.

An example of an oil immersion counterpropagating measurement is shown in the bottom center of Fig. 15. The enhanced spatial resolution is evident with features of 285 nm being easily resolved in the single-frequency IR lipid image at 1740 cm^−1^. In this configuration, the sample must be a thin sample (≲10 *μ*m) and placed on a standard glass cover-slip (typically 170 *μ*m thick) as such oil immersion objectives are designed to be used only with glass coverslips. In addition, the sample is presented upside down, such that the IR excitation, being delivered from beneath in this counter-propagating mode, will strike the sample first without having to be transmitted through a substrate, with associated wavelength transmission and focal dispersion considerations.

One important consideration when using glass substrates, be it in counter-propagating mode or co-propagating mode, is that with thinner samples ≲7–10 *μ*m, as in the case of a single dried cell as shown in Fig. 15, that since the IR beam is passing through the sample and interacting with glass substrate, some broad silicate absorbances, with superimposed IR absorbances from the sample, are evident in the spectral range of ∼1300–800 cm^−1^. For any single frequency imaging, this will need to be factored into appropriate selection of ratio image combinations, but for instances of where the biomolecular spectral features lie outside of this glass region, such as protein and lipid absorbances, such glass substrates are ideal and very practical as they align well with existing typical biological sample preparation workflows.

**FIG. 16.**
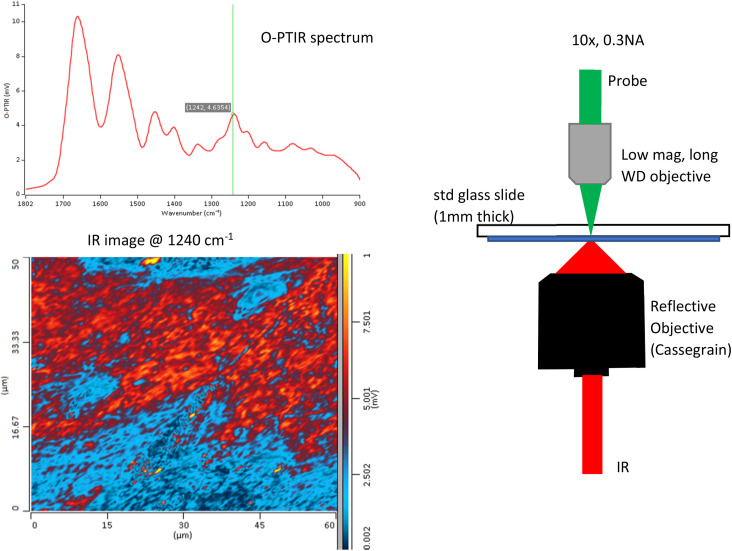
Use of low NA probe beam objective for large area O-PTIR mapping.

Similarly, in Fig. 16 we show the applicability of regular 1 mm thick glass slides as substrates for O-PTIR. Here, a regular histological tissue section has been placed on a standard 1 mm glass slide and measured in counter-propagating mode, but this time with a low magnification, low NA glass objective as only low NA glass objectives provide good, distortion-free imaging when measuring through a relatively thick optical element, such a 1 mm glass slide in this case. The use of a low NA probe objective also provides benefits in terms of a larger measurement spot size (∼1 *μ*m), which is useful when need to image larger FOVs but also in terms of a deeper depth of field, which can be advantageous for tissue sections with higher topological variability. It is worth noting that, in this case, compared to Fig. 15 above, no broad glass silicate features are observed, as in this case, the sample was relatively thick at least 7–10 *μ*m. This means that little to no IR energy is transmitted to the underlying glass substrate, thus no glass features are observed.

### O-PTIR sample thickness/size impacts and sensitivity limits

**FIG. 17.**
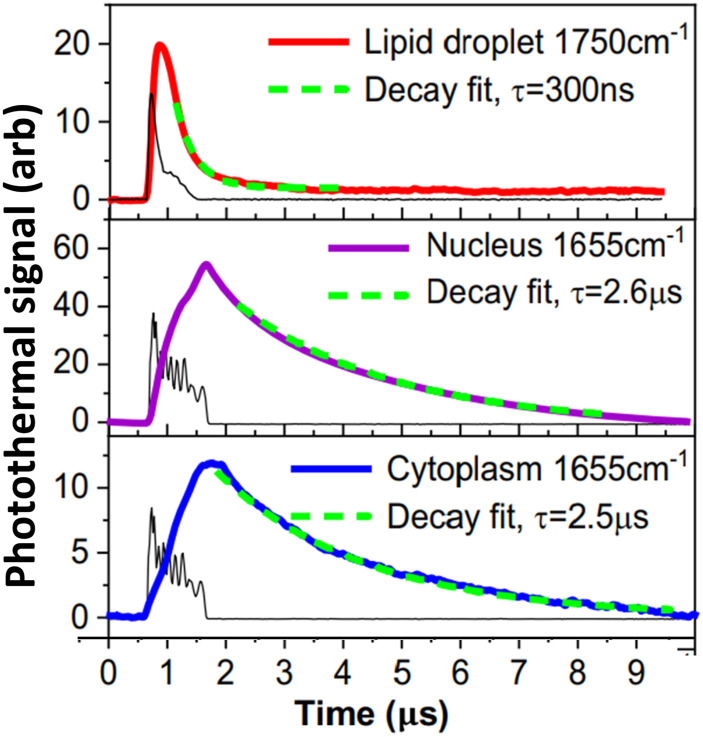
Example peak signal levels and thermal decay times for different cellular components. Colored traces show the time-dependent photothermal response of the cellular components. (Thin black traces show auxiliary measurements of the IR source pulses.) Adapted and reprinted with permission from Yin *et al.*, Nat. Commun. **12**(1), 7097 (2021). Copyright 2021 Author(s), licensed under a Creative Commons Attribution 4.0 License.

Unlike conventional transmission infrared measurements, O-PTIR can measure arbitrarily thick samples when operated in co-propagating mode, whereas transmission IR requires a sample thin enough for IR radiation to transmit through it to a detector. On the other end of the size scale, O-PTIR has also been used to measure very small objects below the detection sensitivity of conventional IR, including polymeric microspheres to 100 nm diameter,^16^ individual bacteria,^16,46–48^ and sub-micron lipid droplets.^22,49^ The measured signal level in O-PTIR typically has a somewhat complex dependence on the size of the object measured in part because the measured signal depends on not only the peak temperature but also the thermal decay dynamics, which impacts the time duration of the photothermal distortion sampled by the probe beam. Smaller objects lose heat more rapidly, and hence, the demodulated photothermal signal can have nonlinear dependence on an object’s size. Li *et al.* have modeled these effects^16^ and example measurements of the thermal decay dynamics of different cellular components are shown in Fig. 17, adapted from the work of Yin *et al.*^22^ Interferometric effects can also enhance or reduce the detection sensitivity based on coherent interference of the probe beam interactions with the sample of interest and with an underlying substrate.^16,50^ The sample thermal diffusivity (which depends on thermal conductivity, heat capacity, and density) also impact the thermal decay dynamics and hence the detected signal level.

### Quantitation

A common question about O-PTIR is whether it can be used for quantitative extraction of component concentrations similar to the use of the Beer–Lambert law in conventional FTIR. The signal in O-PTIR does have a linear dependence on the concentration and molecular absorptivity but has a more complex dependence on the object size due to the effects mentioned above. The O-PTIR signal also depends on several other material properties not included in Beer–Lambert, including the sample density, heat capacity, and thermal conductivity. As such, the O-PTIR signal amplitude itself is generally not used for direct quantitation, but semi-quantitative measurements can be made using ratiometric approaches, e.g., the ratio of spectral band intensities or ratios of O-PTIR absorption images at different wavelengths. For example, one can use the ratio of absorption of a carbonyl ester band to an amide band to obtain a relative measurement of lipid to protein concentration variations. These approaches are discussed in more detail later in this article. Pavlovetc *et al.* have performed systematic tests of the photothermal signal intensity vs radius for polystyrene microspheres and observed a linear dependence on temperature rise and quadratic dependence on radius.^51^

### Detector choice for photosensitive samples

Traditional IR systems (both FTIR and direct QCL based) employ infrared detectors and also often require cryogenically cooled IR detectors (e.g., with liquid nitrogen). Advantageously, O-PTIR is typically performed with one of two different visible, room-temperature photodetectors. For routine work at mW probe power levels, silicon PIN photodiodes are used, which provide excellent SNR and dynamic range. For samples that are highly sensitive to potential photodamage from the visible probe beam, more sensitive detectors such as avalanche photodiodes (APD) or photomultiplier tubes (PMT) can be used. The use of APDs enables O-PTIR measurements at microwatt level probe power levels, which has proven essential, for example, is common for the measurement of dark, colored, or otherwise easily damaged samples, including samples that are difficult or impossible to measure with Raman spectroscopy due to generally higher required excitation power. The use of microwatt level probe power with APD detectors has enabled vibrational spectroscopic measurements with sub-micron spatial resolution on samples that could potentially be damaged by higher excitation, including pigmented plastics,^52^ hair,^53^ polymeric spherulites,^54^ biological cells,^32^ and fresh hydrated tissue.^38^ If when measuring a sample with O-PTIR one observes signal instability and/or morphological or spectral changes in the sample at higher probe powers, the use of lower probe power and APD detection is advised.

### Avoiding sample damage

For most samples, it is possible to find operating conditions to avoid sample damage. Commercial O-PTIR instruments have variable attenuators to permit adjustment of the IR and/or probe power on the sample. In general, the SNR increases with increasing probe and IR power as long as the intensities do not exceed the damage threshold of the sample. When adjusting settings to determine optimal operating conditions, it is useful to perform the following steps.

(1) Acquire optical images before and after O-PTIR spectral measurements to verify that the sample is not visibly altered by the IR and/or probe beams.
(2) Observe the O-PTIR signal intensity at a strongly absorbing band over time and ensure that it is stable. If the O-PTIR signal rises or falls quickly, it is usually an indication that the sample’s damage threshold is exceeded.
(3) Acquire multiple successive spectra from the same location to ensure the spectra are reproducible or observe the co-averaged spectral acquisition, keeping an eye out for any drastic spectral changes from scan to scan, which would indicate likely sample damage. Each scan in a set of co-averaged spectra should help increase spectral SNR only and not result in spectral changes.

If damage/instability is observed, the IR and/or probe power should be reduced until stability and reproducibility are achieved. Dark or colored samples tend to be more easily damaged by the visible probe beam and can be more optimally measured at lower optical power and using APD detectors as described above. In general, organic and polymeric samples may need to be measured at lower IR/probe power, whereas inorganic samples can usually tolerate higher IR/probe powers. Examples of diagnostics for detecting damage and methods to avoid are described by Sandt and Borondics.^53^

### Molecular orientation

**FIG. 18.**
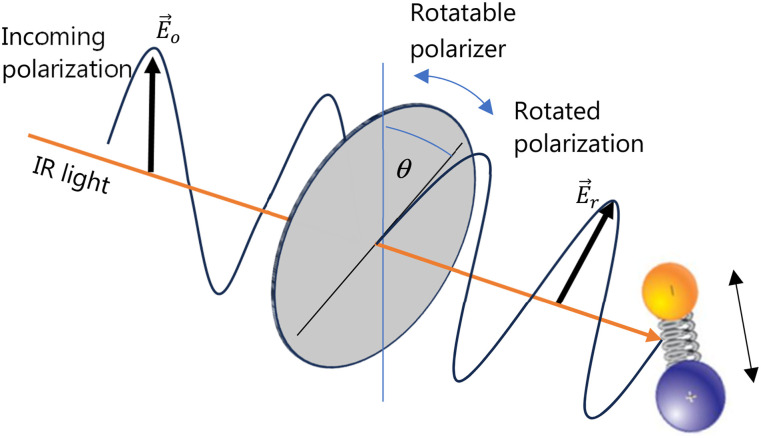
Schematic for polarization sensitive infrared spectroscopy with O-PTIR. The amount of IR absorption is dependent on the relative angle between the electric field of the IR radiation and the molecular bonds in the irradiated molecule, which controls the efficiency of excitation of molecular bond vibrations. Measuring O-PTIR images and spectra at different polarization rotations provides insight into molecular orientation within a sample.

O-PTIR has proven to be a powerful tool for examining molecular orientation in both polymeric^54,55^ and biological^56,57^ applications. Taking advantage of the inherent high polarization of mid-IR laser sources used in O-PTIR, researchers have studied the polarization sensitivity of IR absorption bands to infer details about the molecular orientation of the absorbing bonds. In particular, the efficiency of excitation of a molecular bond is dependent on the relative alignment of the bond axis and the electric field of the incident IR radiation. Measurements of O-PTIR spectra and/or images are usually performed at multiple relative polarization orientations. Early work was done by rotating the sample on the measurement stage, but more recently, polarization rotation capabilities have been integrated into O-PTIR instruments (Fig. 18) to adjust the incident orientation of the electric field of the incident IR radiation on the sample at prescribed intervals. To avoid ambiguity in the extracted orientations, measurements are generally performed at three or more polarization orientations to extract molecular orientation angles.^54,56^

**FIG. 19.**
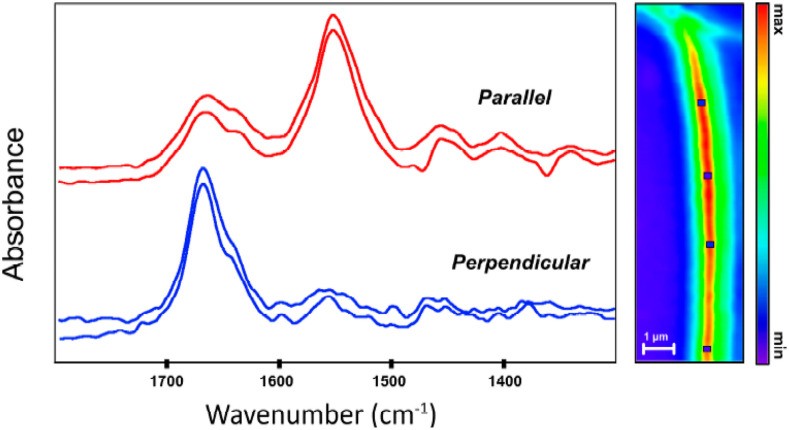
Example of molecular orientation effects observed in O-PTIR spectra measured on a collagen fibril. Reprinted with permission from Bakir *et al.*, Molecules **25**(18), 4295 (2020). Copyright 2020 Author(s), licensed under a Creative Commons Attribution 4.0 License.

**FIG. 20.**
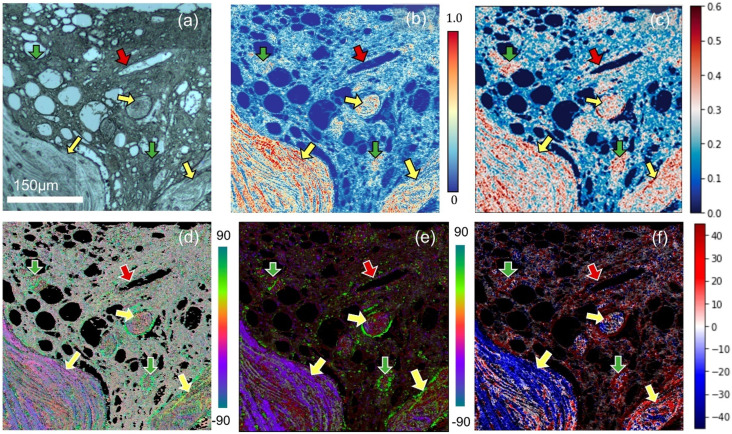
Collagen and its orientation from polarization-sensitive O-PTIR imaging at two wavenumbers (1536 and 1660 cm^−1^); visible image (a) and O-PTIR image (b) at 1660 cm^−1^ shows a region of interest from bone marrow biopsy that includes trabecular bone (yellow arrows), blood vessel (red arrows), and diffused collagen (green arrow). The image in panel (c) shows the polarization sensitivity of each pixel computed from polarization-sensitive O-PTIR images; collagen orientation represented with amide ratios from three polarization as red-green-blue color channels of the image in panel (d) with red: R0°, green: R45°, and blue: R-45°. The image in panel (e) shows principal orientation (angle in degrees) of fibers in this image as indicated by the color bar. The image in panel (f) shows the projection of the tensor onto an angular distribution. Reprinted with permission from Mankar *et al.*, Appl. Spectrosc. **76**(4), 508 (2022). Copyright 2022 SAGE Publications.

**FIG. 21.**
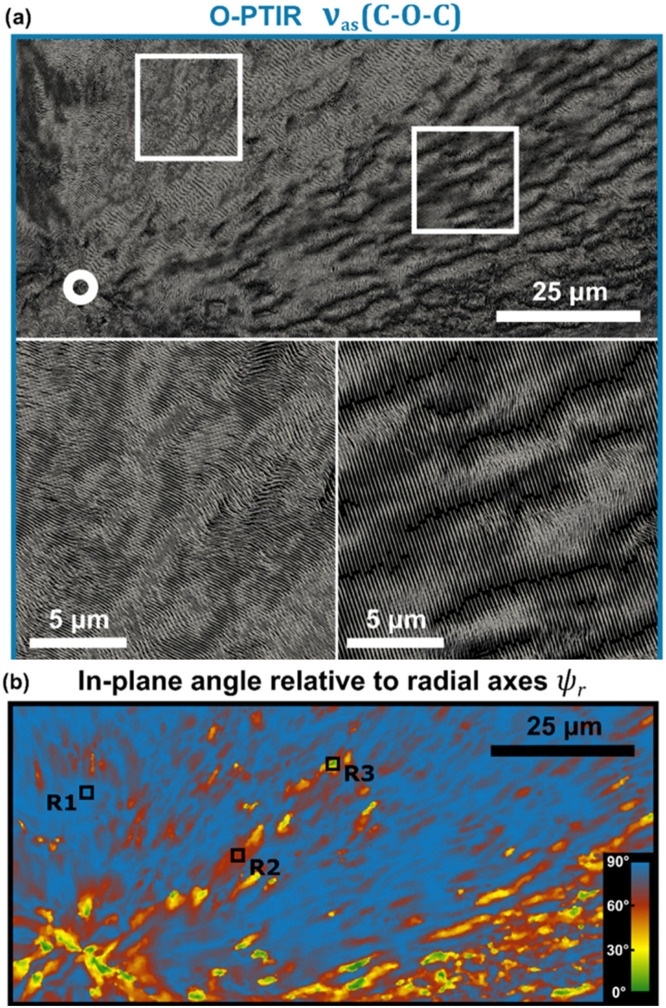
3D mapping of polymer chain orientation in a spherulite. Reprinted with permission from Koziol *et al.*, J. Am. Chem. Soc. **144**(31), 14278 (2022). Copyright 2022 Author(s), licensed under a Creative Commons Attribution 4.0 License.

Figure 19 shows an example of polarization sensitive O-PTIR measurements performed on a collagen fibril showing a reversal in the relative amplitudes of the amide I and amide II protein-associated bands.^57^ Figure 20 shows the measurements extracting the quantitative molecular orientations of collagen in a biological tissue section, and Fig. 21 shows the 3D mapping of molecular orientation in a polymer spherulite.^54^

## SAMPLE PREPARATION TECHNIQUES

Sample preparation for O-PTIR measurements depends on the sample type and can range from immediate measurement with no sample preparation to more complex techniques including labeling, fixing, sectioning, digestion, filtration, and other approaches depending on the sample and analysis requirements. Common sample preparation techniques are described in the following and presented in Table I, along with recommended measurement configurations for each sample type.

**TABLE I.** Example sample types and recommended measurement configurations.

Sample type	Preferred substrate(s)	Substrate thickness	Sample prep	Example reference	Co-prop	Counter-prop	Detection path	Sample orientation	Objective style	Objective mag.	Objective NA	Achievable spatial resolution (nm)
Thin film/tissue sections (<5–10 *μ*m thick)	Glass slides	1–2 mm	Embed, microtome, and dewax	Mankar *et al.*^56^	X		Epi	Upright	Cassegrain	40×	0.75	500–800
	CaF_2_	350 *µ*m		Prater *et al.*^39^		X	Epi	Upright	Refractive	50×	0.8	300–500
Thick tissue sections (>10 *μ*m) or blocks or live hydrated tissues	Glass slide	Any		Gvazava *et al.*^69^	X		Epi	Upright	Cassegrain	40×	0.78	500–800
Dried cells	CaF_2_	350 *µ*m	Culture and fix or drop cast	Spadea *et al.*^35^ Bai *et al.*^25^		X	Epi	Upright	Refractive	50×	0.8	300–500
	Glass slide^a^	1 mm		Kansiz *et al.*^71^	X		Epi	Upright	Cassegrain	40×	0.78	500–800
Live/hydrated cells	Glass coverslip, ∼5 *μ*m spacer, CaF_2_ window	170 *μ*m glass coverslip on top, 350 *μ*m CaF_2_ on bottom				X	Trans	Upright	Coverslip compensated refractive	40×	0.95	300–500
	CaF_2_ window/5 *μ*m spacer, glass slide or coverslip	350 *μ*m CaF_2_ on top, thin or thick glass (up to 1 mm) on bottom		Shuster *et al.*^49^	X		Trans	Cells on inverted CaF_2_ window, glass coverslip beneath	Cassegrain	40×	0.8	500–800
Bacteria/spores and other small (<5–10 *μ*m thick) particulates	Glass^a^	Any	Culture and incubate or drop cast	Xu *et al.*^37^ Lima *et al.*^47–49^	X		Epi	Upright	Cassegrain	40×	0.78	500–800
	CaF_2_	350 *µ*m				X	Epi	Upright	Refractive	50×	0.8	300–500
Microplastic particles	Gold coated polycarbonate filter, Simpore filter	Any	Vacuum filtration	Tarafdar *et al.*^89^			Epi	Upright	Cassegrain	40×	0.78	500–800
Atmospheric particles, drug aerosols	CaF_2_ or glass for >5 *μ*m particles	Any	Atmospheric sampling/fractionation	Khanal *et al.*,^88^ Olson *et al.*^90^	X		Epi	Upright	Cassegrain	40×	0.78	500–800
Thick (>10 *μ*m) and/or opaque samples, bulk samples	Any	Any	None if smooth, cut/polish if needed	Jubb *et al.*^124^	X		Epi	Upright	Cassegrain	40×	0.78	500–800
Polymer multilayers, cultural heritage	CaF_2_ for sections <10 *μ*m, any for >10 *µ*m	Any	Embed in epoxy and cross-section	Marcott *et al.*^116^ Beltran *et al.*^106^	X		Epi	Upright	Cassegrain	40×	0.78	500–800

^a^ Use of glass slides can provide interference with a glass silicate band (∼1300–800 cm^−1^), particularly for samples ≪10 μm thickness, with some biological bands (such as nucleic acids and carbohydrates), but suitable for studies of proteins with a clear access to amide I (for protein secondary structure) and amide II, lipids. For samples >10 μm thickness, glass absorption interference is often negligible across the entire IR spectral region.

### No sample preparation

Many samples require no special sample preparation. If the features of interest occur on the top surface of a sample, many samples can be loaded into the microscope and measured directly. O-PTIR microscopes typically have mounting features for glass slides and other similar sized sample holders and available sample areas of around 100 × 100 mm^2^. In the case of larger samples, it may be necessary to simply cut a portion that will fit on the sample stage. Examples of such samples include thin films/coatings on substrates, polymeric materials, and similar bulk materials.

### Drop casting/spin coating

For many polymeric materials and biological materials such as cell suspensions, drop casting is a simple approach for sample preparation. In this method, the sample suspended or dissolved in a solvent is typically pipetted onto a sample substrate and the solvent is permitted to evaporate. Serial dilutions are often used, e.g., stock solution, 10× dilution, 100× dilution, 1000× dilution to find a concentration that provides adequate coverage without excessive pileup of sample material. To achieve uniform coating thicknesses for polymer films, spin-coating approaches^26^ may be used where the solution is pipetted onto a spinning sample at a given rotation speed to achieve the desired thickness.

### Sectioning/polishing

Sectioning and/or polishing are often used for tissue (sectioning) and geological samples (sectioning/polishing). For tissue samples, sectioning is generally used to extract a slice of tissue from a tumor biopsy or from a specific location in an organ. Such slices are usually created via microtomy and may involve embedding tissue prior to sectioning. It should be noted that most embedding materials (e.g., paraffin, epoxy resins, and nitrocellulose) have strong IR absorption peaks of their own, so it may be desirable to remove the embedding medium prior to chemical analysis by O-PTIR. The use of cryomicrotomy (i.e., freezing the tissue before sectioning) can eliminate the need to add and then later remove embedding material. Cross sectioning can also be useful when studying layered materials, e.g., a polymer laminate film, cultural heritage items such as paintings, and the examination of sub-surface defects. For fine sectioning, microtomy is still preferred, but in some cases, sectioning by a razor blade may produce sufficient results.

### Fixed cells, including fluorescent labeling

For biological cells, sample preparation techniques already commonly used for conventional light microscopy, including fluorescence microscopy, are often directly applicable to O-PTIR measurements. For example, conventional approaches for cell culturing, fixing, and immunofluorescence labeling are all compatible with O-PTIR measurements. Unlike Raman microscopy, O-PTIR measurements are immune to fluorescence interference and in fact fluorescence labeling can be a very powerful technique to help guide O-PTIR measurements to specific regions of interest.^39,58^ Antifade media should generally be avoided in fluorescent labeling of biological samples since common antifade media while optically transparent, often contain strong IR absorption bands that can overlap/interfere with the measurement of biologically relevant IR bands.

For co-propagating measurements, cells can be deposited/incubated on conventional glass slides and cover slips. Plastic bottomed petri dishes are generally not recommended because they have strong IR absorption bands that can overlap with some biologically relevant absorption bands. Zhang *et al*. have used conventional plastic petri dishes with the bottoms replaced with CaF_2_ windows for some measurements of biological samples in aqueous solutions.^11^ For the counter-propagating O-PTIR configuration, cells should be incubated or deposited on IR transparent windows to permit IR excitation light to reach the sample. The most commonly used IR transparent windows that are thin CaF_2_ disks, for example, 10 mm diameter, 0.35 mm thick, e.g., available from Crystran. These CaF_2_ disks can be directly mounted into an appropriate sample holder for either measurements in air or aqueous environments.

In the case of bacteria, aqueous suspensions are often drop cast directly onto an appropriate sample support, e.g., glass slides, coverslips, or CaF_2_ disks and air dried without fixation. For mammalian cells where it is important to preserve the structure of the cell, conventional cell fixation techniques can be appropriate, although with certain caveats. Chemical fixation using cross-linking fixatives, such as formaldehyde or glutaraldehyde, form additional chemical bonds in the sample, which while preserving the cell morphology can introduce conformational changes that can impact the IR spectra. For O-PTIR measurements, where the focus is on detecting specific chemical bonds and maintaining the native chemical state of the sample, alcohol-based fixation (e.g., methanol or ethanol) may be preferred. These fixatives are less likely to introduce new chemical groups and generally cause fewer changes in chemical bond structures compared to aldehydes.

### Sample preparation for live cell measurements

O-PTIR measurements on live cells are generally performed by first culturing cells on a glass or IR transparent cover slip and then mounting the cover slip in a sealed sandwich configuration or in a flow-through fluid cell. Sealed fluid sandwiches can be readily made by cutting a small piece of thin double-sided tape (e.g., 3M 82600^59^), punching a hole in the center and adhering the tape to a glass cover slip to create a small well. A small drop of buffer containing suspended cells can be pipetted into the well and then covered and sealed with an IR transparent window. Depending on the cell type, the cells can remain alive and hydrated for tens of minutes to a few days. For better control of temperature, CO_2_, nutrients, and/or for the introduction of drugs or other bioactive agents, flow-through fluid cells can be used. It is often desirable to limit the thickness of the water layer within the fluid cell to prevent excessive IR absorption by water. In some cases, it can be desirable to use D_2_O (heavy water), which shifts the H–O–H bending absorption band in water away from the amide I band associated with biological proteins.^22^ As will be discussed later, D_2_O has also been used for isotopic labeling of cells, for example, for metabolic studies.

### Measurement and data processing approaches

A key aspect of obtaining informative data is the approach to data acquisition and analysis. For O-PTIR imaging, it is important to understand that the brightness at any pixel is not just dependent on the IR absorptivity, but can also depend on the sample’s thickness, distance from the focal plane, and other thermophysical properties such as heat capacity, density, thermal conductivity, and size, all of which can contribute to the sample’s maximum temperature and how quickly it cools after each IR pulse. As such, the contrast in an O-PTIR image at a single wavenumber may not be representative of the IR absorption only. To overcome this issue, two different approaches are recommended: (1) multi-wavenumber ratiometric images and (2) multi-color overlay images.

### Ratiometric images

Consider an O-PTIR image with a signal intensity *S*(*x*, *y*, *ν*), where (*x*, *y*) are the locations on the sample and ν is the IR wavenumber used to collect the O-PTIR image. This signal can be broken down into two separate functions,

$$S\left(x,y,\nu \right)=A\left(x,y,\nu \right)B\left(x,y\right).$$ (7)

(1) $A\left(x,y,\nu \right)$ which represents the IR absorption as a function of (*x*, *y*) position and wavenumber ν
(2) *B*(*x*, *y*) which represents the other sources of variation in the O-PTIR signal, for example, reflectivity, sample thickness, and thermal/mechanical properties.

Calculating the ratio of the signal *S* at two different wavenumbers ν1 and ν2 gives

$$\frac{S\left(x,y,\nu 1\right)}{S\left(x,y,\nu 2\right)}=\frac{A\left(x,y,\nu 1\right)B\left(x,y\right)}{A\left(x,y,\nu 2\right)B\left(x,y\right)}=\frac{A\left(x,y,\nu 1\right)}{A\left(x,y,\nu 2\right)}.$$ (8)

**FIG. 22.**
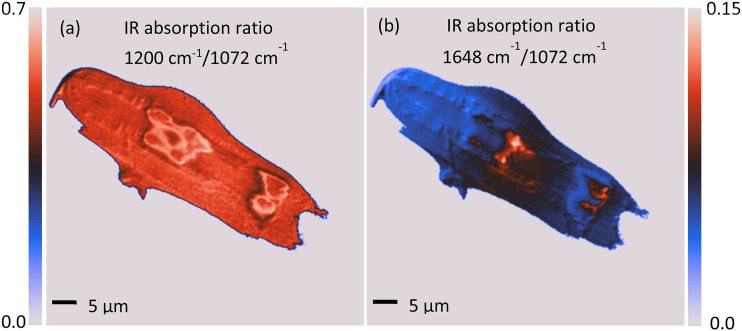
Examples of ratiometric images of a diatom displaying the ratio of IR absorption at different IR bands. (a) Ratio image of two different silica bands; (b) ratio image of protein to silica bands. In both images, the ratio has been set to zero when the absorption signal was below a given threshold (i.e., regions with minimal signal away from the diatom). Wide-field fluorescence-detected O-PTIR images are shown. Reprinted with permission from Prater *et al.*,^45^ Appl. Spectrosc. (published online) (2024). Copyright 2024 The Authors.

In this case, the reflectivity, sample thickness, and thermal/mechanical properties are constant at each point in the sample; thus, the *B*(*x*, *y*) term in the numerator and denominator are the same and cancel out in the ratio. The ratio image thus reveals the variation in IR absorption while eliminating effects from other sample properties. Thus, it is strongly recommended to display ratio images and optionally any single wavenumber images. Example ratiometric images from a widefield fluorescence-detected O-PTIR measurement are shown in Fig. 22.

For single point measurements, ratiometric images should preferably be acquired in an interleaved mode where O-PTIR images are acquired at different wavelengths by alternating between wavelengths at each scan line in the image to avoid any image drift between successive images. Similarly, for widefield O-PTIR measurements it is advisable to acquire images at wavenumbers of interest over a short period of time or otherwise correct for drift to ensure registration between the images in the ratio calculation. When performing a ratio calculation, it can be desirable to employ a minimum denominator threshold to coerce the ratio calculation to zero for regions of the denominator images where there is no appreciable signal. (This avoids the issue of dividing by a number near zero, which could otherwise obscure real features in the image ratio.)

### Multi-color overlay images

Multi-color overlay images can also be a useful way to highlight differences in absorption at different IR absorption bands. Although this approach does not eliminate contrast from non-absorptive variables, it does visually highlight where the absorption is different between different regions in a sample. Multi-color overlays are performed analogously to their common use in fluorescence microscopy, except instead of assigning a different color to each fluorophore band, a different color is assigned to each IR absorption band. Current O-PTIR instruments permit automated collection of a sequence of O-PTIR images at different IR bands, which can then be quickly assembled into an RGB overlay image.

### Hyperspectral data analysis

O-PTIR researchers are increasingly collecting larger hyperspectral datasets, which involve either the measurements of spectra at many locations on a sample or alternately the collection of O-PTIR images at many IR wavenumbers. In the cases of large hyperspectral datasets, multivariate analyses and machine learning approaches can be very helpful. Commercial programs, such as CytoSpec^60^ Eigenvector Research^61^ products, and the open source analysis package Quasar,^62^ all offer extensive capabilities for hyperspectral data analysis, including techniques such as principal component analysis; various clustering approaches, including k-means and hierarchical cluster analysis; and spectral decomposition such as multivariate curve resolution. Such approaches can be useful for analyzing large datasets to generate maps of the distribution of different chemical species and extracting representative spectra. Supervised machine learning approaches can also be used to train models to classify sample regions based on subtle spectral variations, for example, for tissue classification.^63^ Multivariate analysis/machine learning approaches can also be used to determine which absorption bands most significantly discriminate different chemical species/spectral variations within a sample and can be used to dramatically reduce the number of IR bands measured and significantly reduced required measurement time. For example, Gajjela *et al.* demonstrated that O-PTIR imaging at only five wavenumbers combined with deep learning was sufficient to outperform state-of-the-art diffraction-limited conventional IR imaging with up to 235 wavenumbers.^63^ Machine learning approaches have also been used for microplastics identification.^64^

## O-PTIR APPLICATIONS

### Biological and biomedical applications of O-PTIR

Mid-infrared spectroscopy and chemical imaging have proven to be vital tools in the life sciences, offering non-destructive, label-free, and chemically specific insights into biological samples. Infrared spectroscopy enables the identification and characterization of key biomolecular categories, including proteins, lipids, nucleic acids, and carbohydrates. With its improved spatial resolution, O-PTIR significantly enhanced the power of IR spectroscopy by providing detailed spatial distribution and concentration data of these molecules with sub-micron spatial resolution. O-PTIR research in the life sciences facilitates a deeper understanding of cellular processes, disease mechanisms, and the composition of complex biological systems. The following sections summarize recent O-PTIR research in biological and biomedical sciences.

### IR tags

Label-free imaging of the main bio macromolecules, such as proteins, lipids, carbohydrates, and nucleic acids, with infrared spectroscopy have been much touted and demonstrated. However, since IR spectroscopy is a “total” analysis technique, i.e., one where all IR active molecules within the measurement volume will contribute to the spectrum, when specific and lower concentration components, such as metabolites are required to be chemically imaged, interferences from the surrounding higher concentrations biomolecules can make this very challenging.

One approach to increase the specificity of IR spectroscopy is to employ IR tags (also referred to as probes and labels). IR tags can be broadly categorized into two classes: (1) Stable Isotopic labeling and (2) so-called “silent region tags.”

**FIG. 23.**
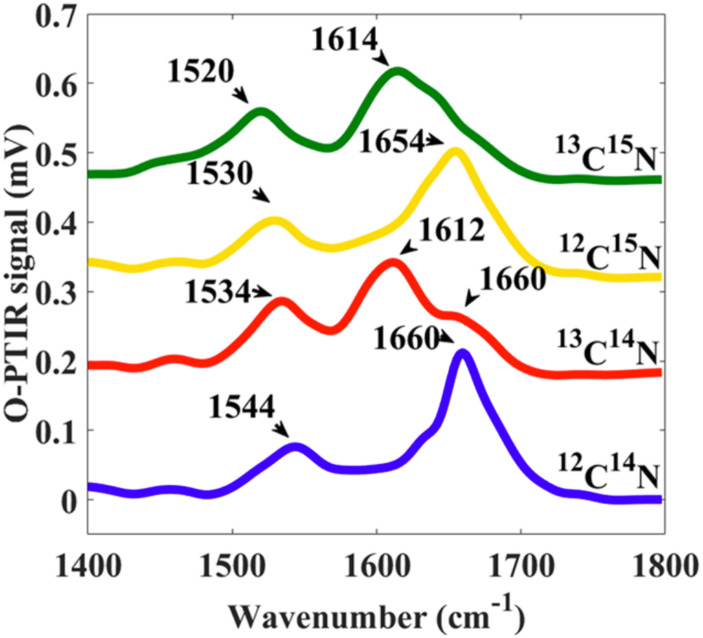
O-PTIR measurements with stable isotope labeling showing amide I and II regions (1400–1800 cm^−1^) on single *E. coli* cells incubated with ^12^C^14^N (blue line), ^13^C^14^N (red line), ^12^C^15^N (yellow line), and ^13^C^15^N (green line). Data reprinted with permission from Lima *et al.*, Anal. Chem. **93**(6), 3082 (2021). Copyright 2021 American Chemical Society.

Stable isotope IR labels most commonly include ^2^H(D), ^13^C, and ^15^N, although, in principle, other stable isotopes will also work. Stable isotopes for increased IR spectroscopic specificity work by generating additional spectral contrast taking advantage of molecular vibrations involving heavier atoms (isotopes) that result in a significant spectral redshift (lower wavenumber) in absorbance of the relevant band. In the case of the C–H vs C–D stretching vibration, as the relative atomic weight difference between H and D is so large, this causes a very large spectral redshift on the order of ∼700 cm^−1^, with C–H stretching vibrations typically at 3000–2800 cm^−1^ and C–D stretching vibrations typically at 2300–2100 cm^−1^, which turn up in the spectral silent region (more on that is provided in the following). With the relative atomic weight differences of ^13^C and ^12^C being much smaller, for protein amide bands, this redshift is on the order of ∼40 cm^−1^, shifting ^12^C labeled amide bands from ∼1655 to ∼1615 cm^−1^ for ^13^C labeled amide bands,^46^ as shown in Fig. 23.

Silent region IR tags deliver specificity and spectral contrast by virtue of their unique spectral features in the so-called infrared “silent region,” typically between 2600–1800 cm^−1^ (see Fig. 1).

Common functional groups with IR active absorbances in this IR silent region include alkynes, nitriles, and azides. While their unique spectral absorbances in the otherwise absorbance-free “silent window” of the spectrum provide the unique spectral contrast, the ability to modify chemicals to incorporate such moieties provides the key element of designer chemical specificity. This chemical specificity is analogous to that of fluorescent labeling where excellent specificity is typically attained, but unlike fluorescent labeling, these IR probes typically have an ultra-low molecular weight, in stark contrast to the often-bulky labels, such as green fluorescent protein (GFP), which has become so commonplace in fluorescence imaging but runs the risk of perturbing normal metabolic functions.

Recent examples of the use of stable isotope labeling from Shams *et al.*^65^ have been applied to bacterial antimicrobial susceptibility testing (AST), where individual bacterial cells were spectrally measured and chemically imaged with O-PTIR. In this example, various *E. coli* strains were grown in dilute concentrations of D_2_O in the presence of antimicrobial agents with the incorporation of deuterium, detected as C–D vibrations, as a marker for metabolism. The benefit of such a single-cell measurement approach is that far less biomass is required, thus greatly speeding up culturing times and thus time-to-answer, which is critical when rapid AST is required. Other examples from Lima *et al.*^46^ demonstrate the applicability of ^13^C and ^15^N labeling via ^13^C labeled glucose and ^15^N labeled ammonium chloride to explore metabolic activity of individual bacterial cells and their interactions within microbial communities.

**FIG. 24.**
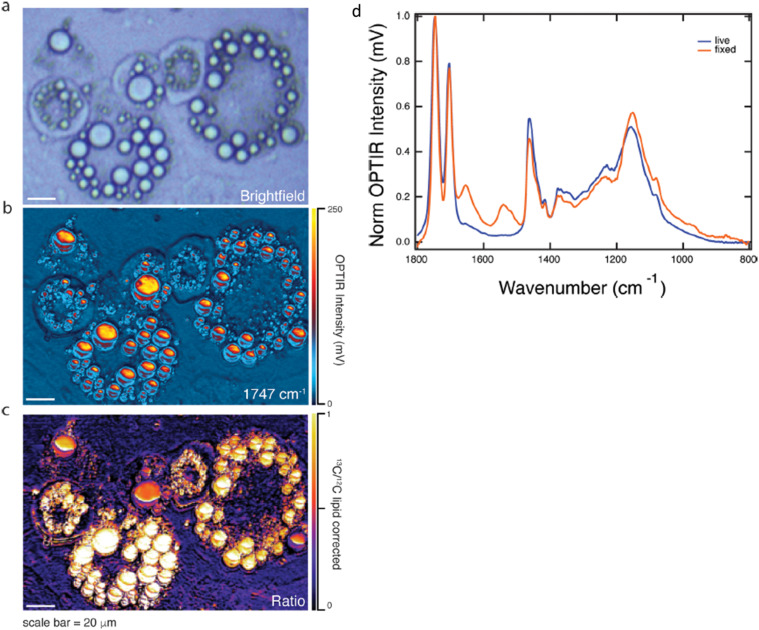
O-PTIR measurements of lipogenesis in live adipocytes 72 h after feeding with ^13^C glucose. (a) Brightfield image. (b) Single wavenumber image collected at 1747 cm^−1^ corresponding with ^12^C lipid ester carbonyl band. (c) Ratio image showing ^13^C lipid ester carbonyl (1703 cm^−1^)/^12^C lipid ester carbonyl (1747 cm^−1^) after correction for amide-I, and water bending band highlighting varying rates of DNL across the cell. Data collected in PBS. (d) Representative spectra from live and fixed cells. Spectra from live (blue) and fixed (orange) differentiated 3T3-L1 adipocytes 72 h after feeding with ^13^C glucose. Reprinted with permission from Shuster *et al.*, J. Phys. Chem. B **127**(13), 2918 (2023). Copyright 2023 American Chemical Society.

Examples of the usage of IR tags for submicron O-PTIR analysis of single and intra-cellular analysis of mammalian (adipocyte) cells have been conducted by Shuster *et al.*^49^ The researchers initially grew cells in regular ^12^C glucose, followed by a switch to ^13^C labeled glucose. This labeled glucose was metabolized and incorporated into the triglycerides in cell lipid droplets, with the ^13^C lipid band appearing at 1703 cm^−1^ compared to 1747 cm^−1^ for ^12^C lipid, providing for a clear peak separation [see Fig. 24(d) below]. The ratio of these two bands, which is correlated to the rate of metabolism, was measured over 72 h. This ratio was also measured using rapid, high spatial resolution single frequency O-PTIR images, providing, both inter- and intra-cellular spatial information on rate of metabolism [see Fig. 24(c) below]. In also a first for the O-PTIR measurements, this experiment was conducted with live, metabolizing cells in water.

A recent example of a silent region tagging with azides yields from Bai *et al.,*^25^ where azide-labeled palmitate was used to investigate the lipid metabolism of single cells from human derived 2D and 3D culture systems. The use of azide labeled palmitic acid provided enhanced sensitivity to lipid detection (including spatial location) of newly synthesized lipids, while the co-located fluorescence imaging modality of O-PTIR proved useful for cell-type identification in complex tissue systems.

Stable isotope labeling has also recently been used for “extraterrestrial” research. Using a reversed ^13^C-stable isotope labeling experiment, Waajen *et al.*^66^ demonstrated the direct metabolic transfer of carbon from an (extraterrestrial) meteorite into microbes, suggesting that organics from meteorites could have been used as a carbon source on early Earth and other habitable planets.

### Neurodegenerative applications of O-PTIR

A very promising application field for O-PTIR has been shown in neurodegenerative disease research. Neurodegenerative diseases can be broadly categorized as diseases where neuronal cells lose function and die over time. Some common examples of neurodegenerative diseases include Alzheimer’s disease (AD), Parkinson’s disease, and Huntington’s disease. While the mechanisms of these various neurodegenerative diseases are complex and different, they often share a common feature— protein misfolding and aggregation. In addition, herein lies the unique fit that O-PTIR possesses for their study, with O-PTIR delivering both the chemical specificity for determination of the protein secondary structure (and misfolding), and uniquely, by virtue of its submicron spatial resolution, it can also locate and resolve these very small aggregates at physiologically relative spatial scales. The protein secondary structure has long been studied with infrared spectroscopy, and it is well known as a powerful tool for absolute or relative protein secondary structure determination with key protein bands having characteristic peak positions and shapes that are directly related to the proteins secondary structure(s).^67^

**FIG. 25.**
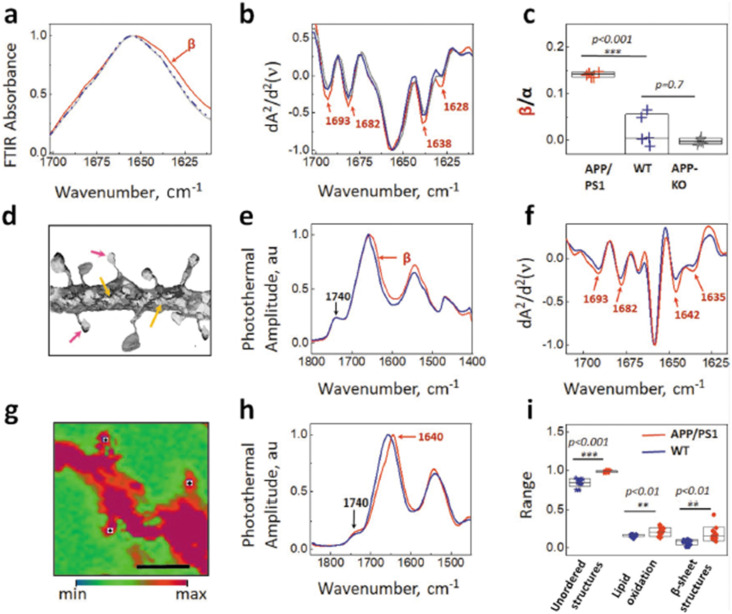
O-PTIR imaging of Aβ aggregates in AD transgenic neurons highlights distinct β-sheet enriched structures, illustrating the technique’s capability to reveal subcellular localization and structural polymorphism of amyloid aggregates in neuronal disease progression. Reprinted with permission from Klementieva *et al.*, Adv. Sci. **7**(6), 1903004 (2020). Copyright 2020 Author(s), licensed under a Creative Commons Attribution 4.0 DEED License.

Initial work in this field by Klementieva *et al*.^68^ focused on the application of O-PTIR on the study of AD-related amyloid protein aggregation directly in neurons of transgenic mouse models at sub-micron, sub-cellular resolution. In a measurement, first, amyloid protein aggregates were detected in neuronal dendritic spines and neurites, leading to a conclusion that amyloid structures exist in different polymorphic forms, with implications in the possibility of different AD progression mechanisms (Fig. 25).

**FIG. 26.**
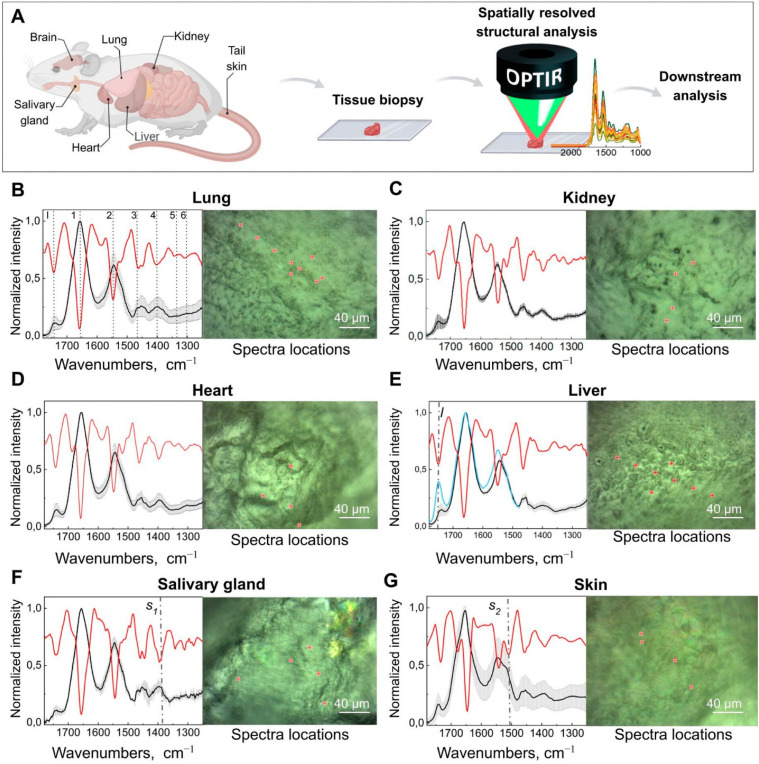
O-PTIR imaging of diverse fresh, hydrated tissue biopsies with submicron spatial resolution capabilities reveal distinct biochemical compositions within unprocessed living tissues. Reprinted with permissions from Gvazava *et al.*, J. Am. Chem. Soc. **145**(45), 24796 (2023). Copyright 2023 Author(s), licensed under a Creative Commons Attribution 4.0 License.

In recent work, Gvazava *et al.*^69^ have taken the study of neurodegenerative diseases related protein aggregates into more biologically relevant conditions with the first ever reports of *ex vivo* and *in vivo* measurements of living, hydrated tissues with infrared micro-spectroscopy (Fig. 26). Freshly extracted tissues, unprocessed and fully hydrated tissues from various organs, such as living brain and lung tissues, and *in vivo* measurement of living salamander embryos were conducted with submicron O-PTIR spatial resolution. The formation of newly formed amyloid aggregates over time in functioning brain tissues was observed for the first time.

Here, it is remarkable that despite conventional wisdom that protein measurements and in particular protein secondary structure determination in the presence of water is impossible, due to overwhelming water absorbances, O-PTIR has demonstrated a unique and valuable property—that of minimal water interferences. Such a capability is invaluable in the measurement of biological systems as it now promises the ability to measure biochemistry under far more physiologically relevant, hydrated, living conditions. Traditional infrared spectroscopy systems, such as FTIR and direct QCL based systems, will suffer significantly from the strong absorbances of water, which almost perfectly overlaps with the key secondary structurally specific amide I band of proteins.

The unique property of O-PTIR of limited water interferences can be essentially attributed to the fact that the mechanism of IR signal detection is very different between traditional IR spectroscopy systems and O-PTIR. In O-PTIR, since it is differential heating from wavelength specific IR absorbance that is being detected, and not the absorbance of IR light directly, like in traditional IR, the fact that water has a relatively high heat capacity compared to biological tissues, such as muscle,^70^ means that O-PTIR is in effect less sensitive to the presence of water relative to other biological materials.

### Measurement of biological samples on histopathology glass slides

Kansiz *et al.*^71^ demonstrated a potential key enabler of O-PTIR toward a more clinical translation by using regular 1 mm thick glass slides as substrates for the measurement and differentiation of different cancerous cell lines. Since O-PTIR operates in reflection mode, as the most commonly employed mode, transmission of IR light through the substrate is not a consideration like it is with traditional IR instrumentation. Furthermore, as O-PTIR reflection mode operation generates regular, FTIR transmission/ATR-like spectral quality, devoid of any dispersive (Mie) scattering artifacts, collected spectra are highly reproducible, which is a critical prerequisite for the detection of the sorts of subtle spectral differences that are found in different cell lines. In contrast, traditional FTIR/QCL based systems almost never operate in direct reflection mode due to poor IR reflection off tissues and cells and the previously mentioned scattering artifacts. FTIR and QCL microscopes are often operating in a “transflection” mode by depositing samples on an IR reflective substrate made from low-e glass. In practice, this approach is a double-pass transmission measurement that can still suffer from IR standing wave effects and Mie scattering artifacts. While some algorithms exist for addressing these artifacts, the O-PTIR approach does not require such spectral corrections.

The high-quality, reproducible reflection mode O-PTIR operation thus means that glass slides are now compatible substrates, effectively meaning that standard clinical workflows and practices can be employed, further enhancing the techniques clinical translatability. In contrast again, a traditional FTIR/QCL system would operate often in transmission mode, requiring IR transparent substrates, such as CaF_2_, which are relatively expensive and fragile. Kansiz *et al.* compared various spectral pre-processing techniques (second derivatives vs no derivatives), modeling techniques (K nearest neighbor vs random forests), and spectral ranges (fingerprint vs C–H region). Classification accuracy of up to 99% was achieved across three different cancerous cell lines using a combination of second derivation spectral pre-processing, random forest modeling with a combined C–H spectral region and fingerprint region.

### Cell measurements in water

**FIG. 27.**
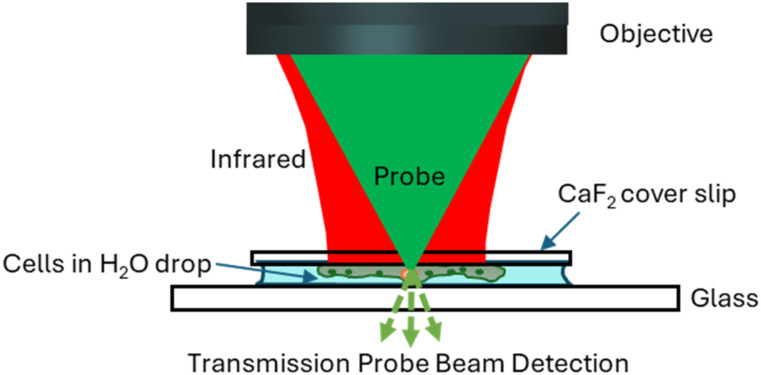
O-PTIR measurement in transmission mode on a glass slide.

A growing body of publications is emerging, utilizing a key differentiating aspect of O-PTIR over traditional IR techniques—the ability to measure cells in water, even live, metabolizing cells. As discussed above, under within the stable isotope labeling section, Shuster *et al.*^49^ demonstrated a first, by measuring the metabolic update of glucose in living, metabolizing cells. In this case, as detailed above, the example from Gvazava *et al.*,^69^ lipid droplets were measured in living salamander embryos with little to no water interferences despite the cells being present in water. This is because the heat capacity of lipids is even lower than that of muscles compared to water; thus, the effective thermal chemical selectivity toward lipids over water is greater. A key instrument mode and configuration consideration for hydrated cell measurements with O-PTIR is that unlike most samples, which have appreciable probe beam reflectivity, the reflectivity of the probe beam (commonly a 532 nm laser) is extremely low due to the existence of very little refractive index differences between the cell and water. In this case, the preferred mode of detection is not reflection, but transmission detection. It is important to reiterate here that unlike traditional IR techniques, discussions of reflection vs transmission detection are with respect to the probe beam, not the IR pump beam. With co-propagating transmission detection, both the IR pump beam and the probe beam are delivered from above, thus necessitating an IR transparent upper cell window. Thin CaF_2_ windows here are ideal, with thicknesses of 350 *μ*m delivering excellent IR transmission with a sufficient visible imaging performance. The transmitted probe beam passes through the lower window (which only now must be transparent to the visible probe beam, thus being compatible with regular glass) before being detected by a dedicated transmission detector. In this configuration, to minimize water absorbances and attenuation, the biological cell is ideally placed on the under-side of the upper CaF_2_ window in a so-called “upside-down sandwich” configuration. As shown in the diagram in Fig. 27, Shuster *et al.*^49^ used ∼5 *μ*m double-sided tape as both a seal and spacer.

Other examples of the measurement of cells in water can be found in the work of Spadea *et al.*^35^ where simultaneous (concomitant) IR (O-PTIR) and Raman spectra were measured in an upside-down sandwich cell configuration to explore and demonstrate subcellular structures such as protein secondary structures and lipid inclusions could be chemical (spectrally) characterized and chemical imaged in water. Shaik *et al.*^72^ published results using fixed cells in water (upside-down sandwich configuration) to demonstrate that various cancerous cell lines could be trained and differentiated while fully hydrated with up to 95% classification accuracy.

### Biomineralization studies

O-PTIR has been used successfully in the study of calcified tissue, including studying bone composition,^73^ growth,^74,75^ and regeneration.^76,77^ Kim *et al.*^74^ used O-PTIR imaging to analyze microcavities within developing synovial joints of mouse embryos, mapping carbohydrate and protein contents at the micron scale within these microstructures. The high-resolution imaging of O-PTIR was crucial for examining the biochemical environment in developing joints, showing its potential to reveal early joint cavitation processes. Ahn *et al.*^75^ used O-PTIR to assess age-dependent changes in the matrix/mineral ratio within mouse femur bone tissue, revealing variations in mineralization patterns as bone tissue matures. Chen *et al.* have used O-PTIR to study the bone healing processes in a rat model involving critical-size calvarial bone defects.^76^ O-PTIR enabled detailed visualization and quantification of the bone's organic and inorganic content, revealing the spatial distribution and relative amounts of key components such as collagen and mineral phases, providing insights related to understanding the effectiveness of different biomaterials in promoting bone regeneration and integration with the host tissue.

**FIG. 28.**
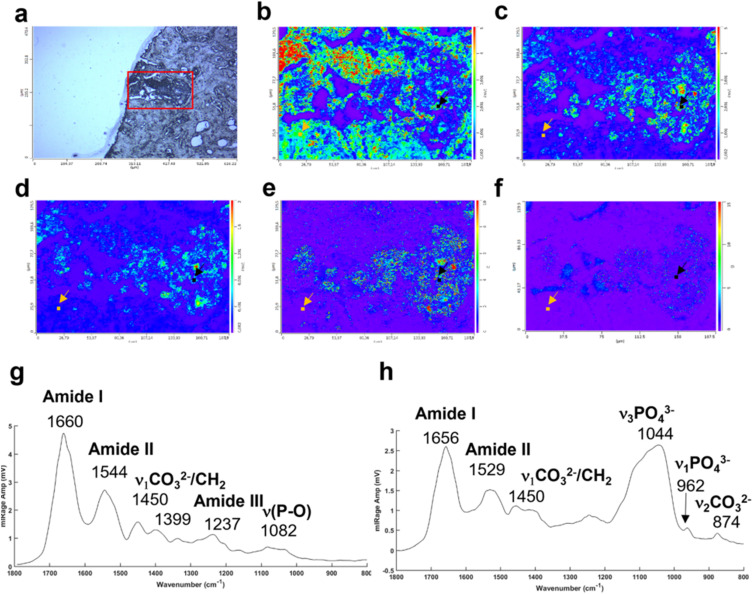
O-PTIR measurements of a breast tissue section, including microcalcifications. (a) White light image (×10 objective) showing a microcalcification in dark (red box). Single IR frequencies collected at (b) protein-associated amide I band (1656 cm^−1^), at (c) phosphate band (1044 cm^−1^), and (d) carbonate band (872 cm^−1^). Panels (e) and (f) show ratiometric images comparing intensity ratios for different bands: (e) phosphate to amide I ratio (1044: 1656 cm^−1^) and (f) carbonate to amide I ratio (872: 1656 cm^−1^). (g) Example of O-PTIR spectra collected from the tissue (orange dot, orange arrow) and (h) within the microcalcification (black dot, black arrow). Reprinted with permission from Bouzy *et al.*, Anal. Methods **15**(13), 1620 (2023). Copyright 2023 Author(s), licensed under a Creative Commons Attribution 3.0 DEED Unported License.

O-PTIR has also been used to study biomarkers associated with bone disease. Clarke *et al.*^78^ used O-PTIR to discriminate osteoarthritic extracellular vesicles (EVs) from those of healthy controls in equine plasma with a classification accuracy of 93.4%, demonstrating a potential use as a diagnostic tool. O-PTIR has also been very successful studying biogenic crystalline disorders, i.e., conditions causing abnormal crystal formations in biological tissue, including breast tissue calcification,^79,80^ arterial calcification,^81^ kidney stones,^80^ and cystinuria.^82^ Figure 28 shows example O-PTIR measurements from Bouzy *et al.*^79^ on breast tissue, where ratiometric images reveal the spatial distribution of carbonates and phosphates, and localized spectra reveal the differences between normal and calcified tissue.

### Biomaterial coatings

O-PTIR has also been used to provide insights into the enhancement of coatings for biomaterials, particularly targeting improved resistance to biofouling and corrosion.^83,84^ Zhao *et al.*^83^ used O-PTIR to assess the effectiveness of bionic engineered protein coatings in preventing biofouling in complex biological fluids with applications related to biomedical devices. O-PTIR measurements enabled the high-resolution visualization and quantification of biofoulant distributions on treated surfaces, demonstrating that the coatings significantly reduce biofouling by repelling biological contaminants. The same research group also used to characterize the dual-protection coatings on magnesium-based biomaterials inspired by tooth enamel biomineralization.^84^ The measurements facilitated the understanding of how these coatings enhance anticorrosion and antifouling properties by providing detailed insights into the molecular interactions at the coating interfaces related to anticorrosion and antifouling functionalities. O-PTIR has also been used to study biomaterials coatings on surgical sutures with the goal of reducing post-surgery infection. Puthia *et al.*^85^ used O-PTIR spectroscopy to analyze the interaction between TCP-25 peptides and polyglactin suture fibers. The O-PTIR measurements revealed that the peptide was successfully integrated into the suture fibers, as evidenced by spectral analysis, which detected specific infrared signatures characteristic of TCP-25 on the suture material. This integration is significant because it enables the sutures to function both as an antimicrobial and an anti-inflammatory agent, enhancing their utility in preventing surgical site infections and reducing inflammation directly at the wound site.

### Pharmaceutical applications

**FIG. 29.**
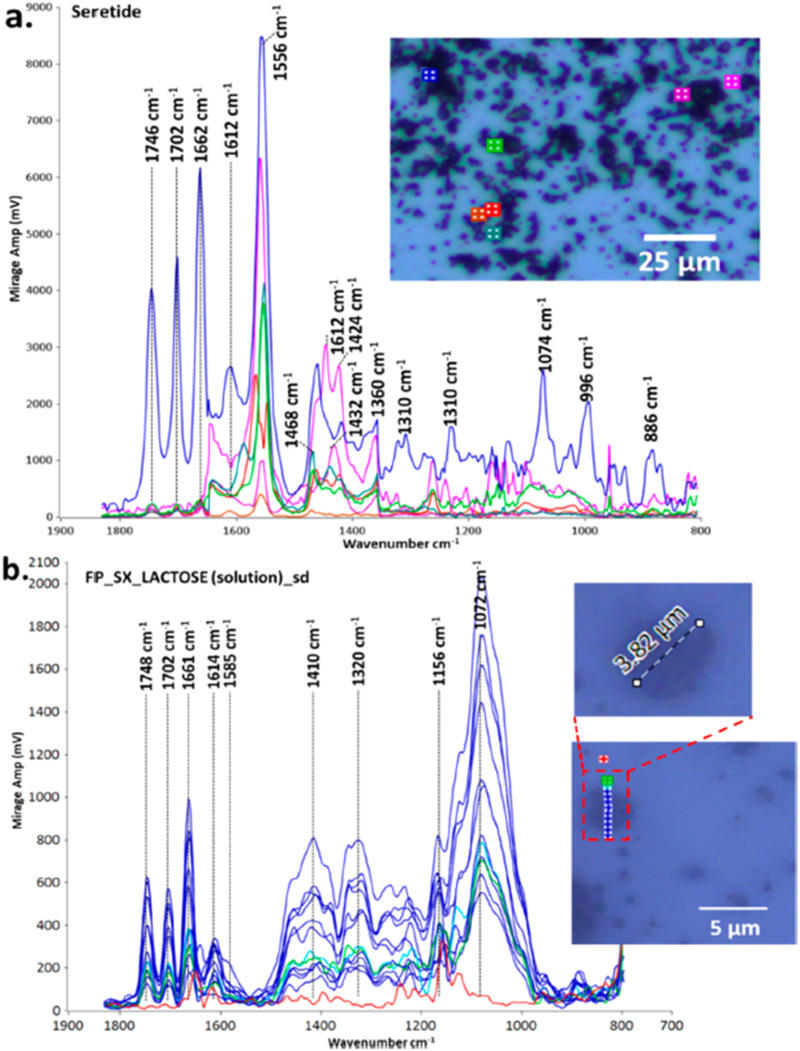
O-PTIR spectroscopic measurements of drug particles from a dry powder aerosol. Reprinted with permission from Khanal *et al.*, Anal. Chem. **92**(12), 8323 (2020). Copyright 2020 American Chemical Society.

O-PTIR has also been used in pharmaceutical research, including mapping of active pharmaceuticals in a drug tablet,^12^ investigating polymer/drug phase separation in amorphous solid dispersions,^86^ and studying pharmaceutical dry powder aerosol formulations,^87,88^ e.g., for asthma inhalers. Yang *et al.*^86^ employed O-PTIR to examine the impact of surfactants on amorphous–amorphous phase separation in drug–polymer mixtures. O-PTIR provided detailed images and chemical composition analyses of phase-separated films, identifying how different surfactants alter the microstructure, which is relevant for understanding drug release and stability in pharmaceutical formulations. As shown in Fig. 29, Khanal *et al.*^88^ demonstrated that O-PTIR can be used to effectively characterize the chemical composition and distribution of drugs and excipients in size-segregated particles of pharmaceutical dry powder inhalation formulations, providing a new analytical capability for evaluating and enhancing drug delivery systems. O-PTIR allowed for the assessment of the spatial distribution of drugs and excipients, suggesting that this technique can provide detailed insights into the colocalization of these components within the aerosol particles. Razumtcev *et al.* employed autofluorescence-detected photothermal infrared to determine the spatial distribution and chemical composition of active pharmaceutical ingredients (APIs) within pharmaceutical mixtures, allowing for detailed characterization of drug formulations, including high-resolution, selective imaging of APIs, with the potential to enable measurements of content uniformity and quality in pharmaceutical products. Recently, studies have also been undertaken to evaluate the ability of O-PTIR to characterize and identify “subvisible” particles, i.e., particles in the range of 2–100 *μ*m. Regulatory guidelines, such as those by the United States Pharmacopeia (USP) and the European Medicines Agency (EMA), require monitoring and controlling subvisible particles, especially in injectable drug products. While existing methods such as light obscuration/flow imaging microscopy can detect subvisible particles and characterize size and shape, the existing analytical methods do not provide information regarding the chemical composition of the particles. O-PTIR has demonstrated the ability to characterize and uniquely identify the composition of subvisible particles,^89^ providing critical data to help ascertain particle sources with the potential for reducing or eliminating the sources of contamination.

**FIG. 30.**
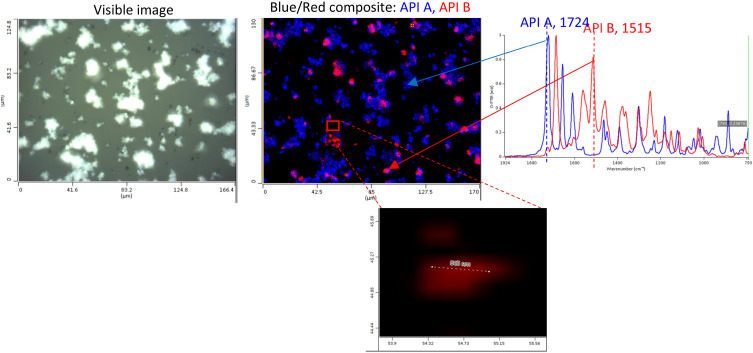
O-PTIR measurement of pharmaceutical inhaler drug formulation dispensed directly onto a glass slide. IR absorption images at IR bands unique to the different APIs reveal the spatial distribution and size of the API particles, with the ability to detect and characterize particles as small as ∼600 nm.

Figure 30 shows O-PTIR spectroscopy and chemical mapping of a binary API inhaled drug formulation sprayed directly onto a glass slide. The resulting O-PTIR spectra clearly demonstrate the high-quality, FTIR transmission/ATR-like spectra, despite being collected in reflection mode off a glass substrate. The spectra show no dispersive scatter artifacts, which in turn translates to high-quality, single IR frequency chemical images on the same sample. By identifying key spectral markers for the compounds of interest, in this case, the two active pharmaceutical ingredients (APIs), rapid, high resolution chemical images can be collected in minutes, depicting an accurate chemical distribution of APIs or other target compounds.

### Environmental applications/microparticle analysis

O-PTIR has been very successful in the field of environmental analysis, especially as associated with detection and characterization of microparticulates, for example, airborne particles resulting from pollution,^90^ sea spray,^91^ and environmental microplastic particles. Similar analysis has been performed on aerosol drug particles, as described above in the pharmaceutical applications section.

#### Microparticle sample preparation

For microplastics and airborne particle sampling, filtration and/or segmentation is often used to prepare samples for O-PTIR. For particles originally suspended in water or other aqueous solutions (e.g., environmental waterway samples and beverages), particles are usually filtered via a vacuum flask using serial filtration, e.g., a first filtration step with 20 *μ*m pores to collect 20+ *μ*m particles and then a second filtration step with 2 *μ*m pores to collect 2–20 *μ*m particles. Metal-coated filters are often preferable over uncoated polymeric filters because polymeric filters will have strong IR absorption bands that can provide an excessive background spectrum when trying to characterize small particles. Gold-coated polycarbonate filters are often a good choice as the thin gold coating blocks IR absorption from the underlying polycarbonate. Inorganic filters such as those etched from silicon materials (e.g., SiMPore filters) can also be an appropriate choice. For certain particle samples such as extraction of microplastic particles from soil or aquatic animal tissue require additional separation/digestion to remove unwanted non-polymeric materials (e.g., inorganics particles and organic tissue) prior to aqueous filtration.

Filtration of airborne particles (e.g., pollution, sea spray, and aerosol drug particles) can be filtered using aerosol particle samplers, which often include size fractionation capabilities based on particle aerodynamic properties. Microanalysis particle samplers have been used to sample airborne pollutants ^90^ and sea spray particles.^91^ Cascade impactors, for example, the Next Generation Impactor^92^ have been used to capture and fractionate samples for O-PTIR.^87,88^

#### Microplastics

Microplastics have been a rich application area for O-PTIR for several reasons. First, O-PTIR provides better spatial resolution and sensitivity than conventional IR micro-spectroscopy, including FT-IR and QCL microscope approaches. A recent publication demonstrated that O-PTIR achieves a much lower limit of detection than QCL-IR and detected 18 times more microplastic particles than QCL-IR^93^ and enabled detection of much smaller particles. Second, O-PTIR avoids the dispersive size/shape/wavelength dependent scattering artifacts. O-PTIR also has advantages over Raman-based approaches as it generally has better sensitivity and is immune to fluorescence interference, which is common in plastic materials. In addition, since O-PTIR can be combined with simultaneous Raman analysis, it can provide additional discriminatory or confirmatory analysis^27,52^ when the particle autofluorescence is not excessive. For these reasons O-PTIR has seen very strong publication growth in microplastics analysis, including detection and analysis of microplastic particles in beverages,^94,95^ food,^96^ baby bottle nipples,^97^ intravenous fluid delivery systems,^89^ bakeware,^93^ aquatic life,^98^ sediment,^99^ and human tissue.^100^ O-PTIR has also been used to analyze chemical changes in microplastic particles associated with photodegradation^101^ and tire wear.^95,102^

**FIG. 31.**
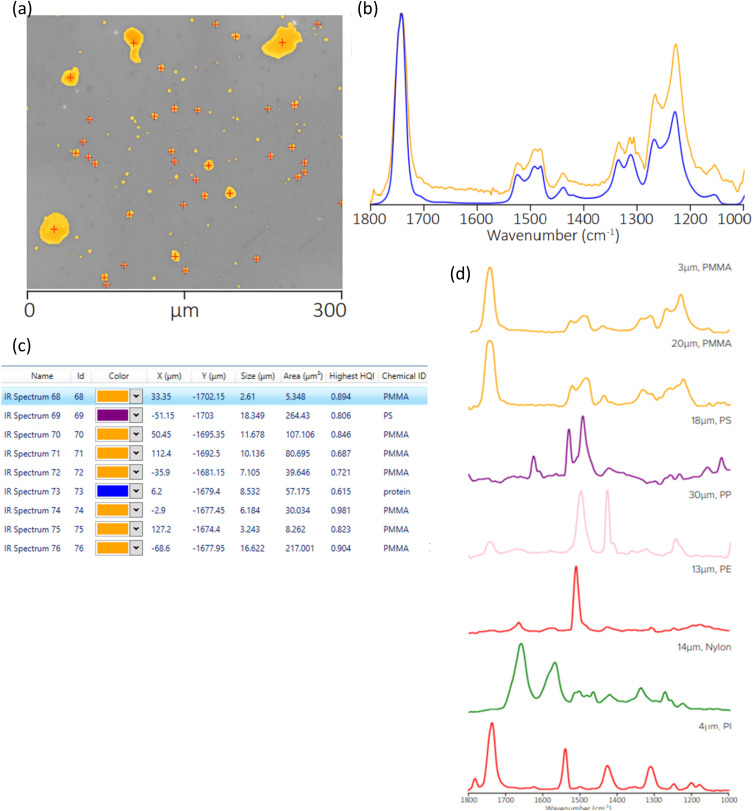
Automation detection of microplastic particles via O-PTIR. (a) Blob analysis showing particle locations, (b) example particle and reference spectra, (c) measured particle summary and chemical identification, and (d) example spectra and chemical identifications of microplastic particles in the 3–30 *μ*m range filtered from water.

While early O-PTIR microplastics measurements were performed manually,^103^ automated analysis of microplastics has recently become available, as shown in Fig. 31. In these approaches, so-called “blob analysis” is first performed on optical microscope images to characterize particle sizes and locations within a field of view. Next, particles may be selected for automated analysis based on size/shape parameters to create a location list for O-PTIR spectral measurements. After automated acquisition of O-PTIR spectra at target locations, O-PTIR spectra are compared against a reference database to provide a chemical assignment for the measured particles.

#### Cultural heritage

Chemical analysis of cultural heritage items via vibrational spectroscopy has a rich history.^104,105^ O-PTIR has been able to extend these analysis capabilities and has been used to analyze historical paintings^106–109^ and decorative objects.^110^ For example, researchers at the University of Antwerp, the Kröller–Müler Museum and SOLEIL Synchrotron used O-PTIR to perform complete stratigraphic analysis of a tiny fragment of a Van Gogh painting^106^ to detect and identify specific pigment particles used in the painting, enabling successful analysis where other techniques had failed. O-PTIR has also been used in the analysis of degradation products in historic painting^107,108^ and glass-metal decorative objects^110^ as well as used as part of a suite of tools to aid in authentication testing of historic paintings.^109^ Marchetti in Ref. 109 noted, “The high spatial resolution (≈450 nm), lack of sample preparation, and comparability of the spectral results to traditional Fourier transform infrared spectroscopy make it a promising candidate for the analysis of cultural heritage,” and “The results clearly demonstrate how O-PTIR can be easily implemented in a noninvasive multi-analytical strategy for the study of heritage materials, making it a fundamental tool for cultural heritage analyses.”

#### Forensics

**FIG. 32.**
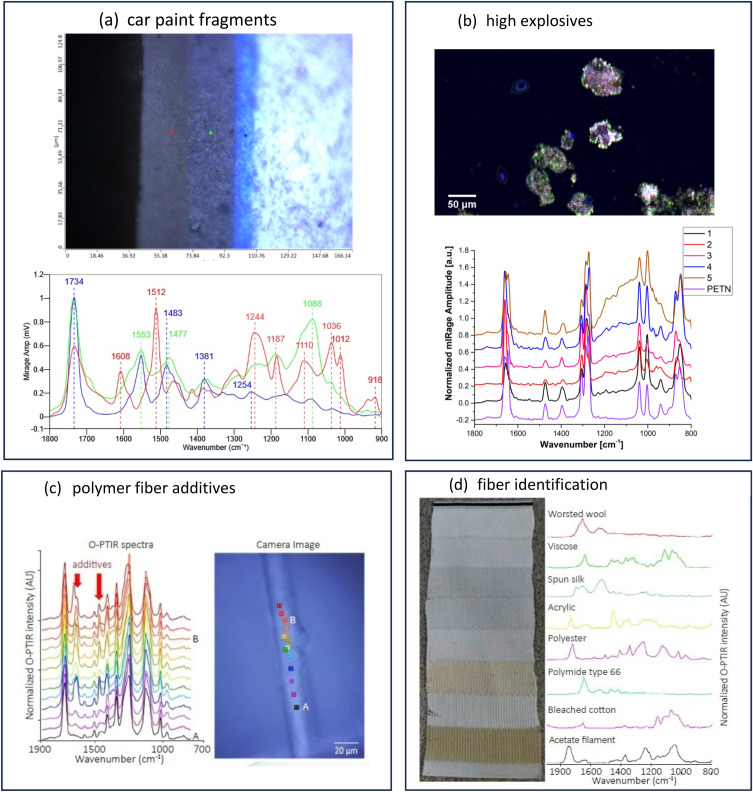
Forensic applications of O-PTIR analysis. (a) Spectroscopic identification of paint/coating layers from automotive paint fragment. (b) O-PTIR characterization of individual explosives particles. (c) Detecting/mapping of polymer fiber additives. (d) Discrimination of different polymer and natural fibers in a forensics fiber reference sample. (a), (c), and (d) courtesy of Photothermal Spectroscopy Corp.; (b) reprinted with permission from Banas *et al.*, Anal. Chem. **92**(14), 9649 (2020). Copyright 2020 American Chemical Society.

O-PTIR also shows promise for applications in forensic sciences. O-PTIR has been used for proof-of-concept demonstrations regarding the identification of paint layers in automobile paint fragments,^111^ characterization of explosives particles,^112^ and successful identification and discrimination of different polymer and natural fibers, including the ability to identify and localized fiber additives (Fig. 32).

#### Materials science

O-PTIR has been used extensively in materials science research, including 2D materials, polymers and composites, battery research, catalysis, and other areas. Example applications are summarized in the following sections.

#### 2D materials

He *et al.* have used O-PTIR microscopy to characterize the chemical distributions within self-assembled microdomains containing amine-functionalized graphene nanoplatelets in a commercial epoxy blend.^113^ Yoo *et al.*^114^ used O-PTIR alongside UV–Vis absorption spectroscopy to analyze functional group inhomogeneity in graphene oxide (GO). By correlating these techniques, the research team was able to identify and map the distribution of different functional groups, particularly C–H and C–O rich regions and examine their electronic behaviors. This dual-spectroscopy approach allowed for a more detailed understanding of how these functional groups affect the electronic and optical properties of GO, informative for optimizing its use in various applications, such as energy storage and biosensors.

#### Polymers and composites

O-PTIR can be a very productive tool for studying polymeric systems because most polymers have both strong IR absorption bands and distinct IR spectral fingerprints that provide robust characterization and identification. O-PTIR has been used to study multi-layer polymer laminates,^115^ including biopolymers^116^ and polymer composites.^26,113,117,118^

**FIG. 33.**
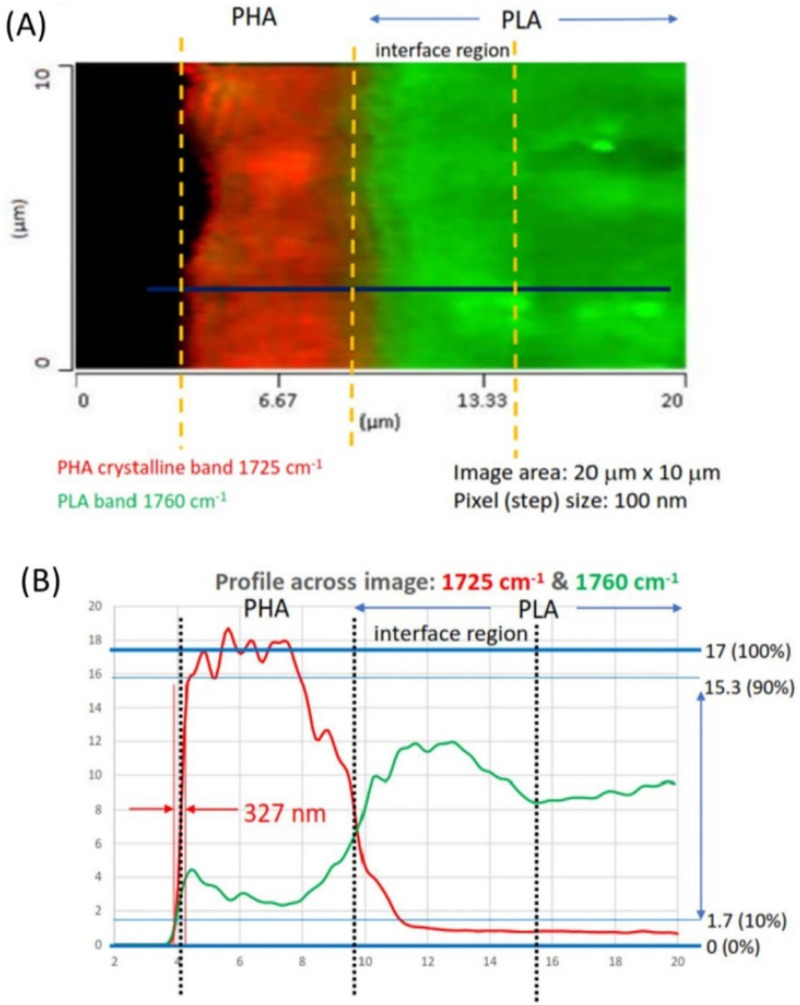
O-PTIR analysis of a polymer multilayer film comprised of degradable bioplastics. Reprinted with permission from Marcott *et al.*, J. Mol. Struct. **1210**, 128045 (2020). Copyright 2020 Elsevier.

**FIG. 34.**
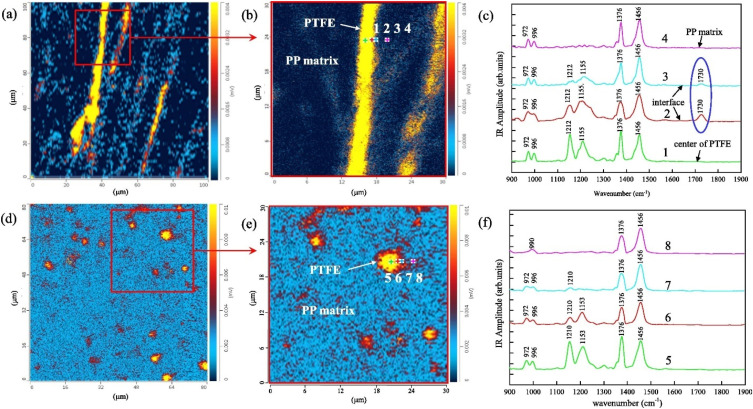
O-PTIIR chemical imaging and spectroscopy of PP/PTFE polymer composite. Panels (a) and (b) and (d) and (e) show IR absorption images on different polymer composite formulations at 1210 cm^−1^ corresponding to an absorption band of PTFE. Panels (c) and (f) show spectra at locations indicated in panels (b) and (e). Reprinted with permission from Zhang *et al.*, Mater. Des. **211**, 110157 (2021). Copyright 2021 Author(s), licensed under a Creative Commons Attribution 4.0 License.

Figure 33 shows an example of analysis by Marcott *et al.*^116^ over the interface between two degradable polymers based on imaging at IR bands unique to the constituents. Figure 34 shows the use of O-PTIR for mapping polymer constituents in a polytetrafluoroethylene/polypropylene (PTFE/PP) reinforced polymer composite.

### Environmentally controlled (heating/cooling/gases) measurements

**FIG. 35.**
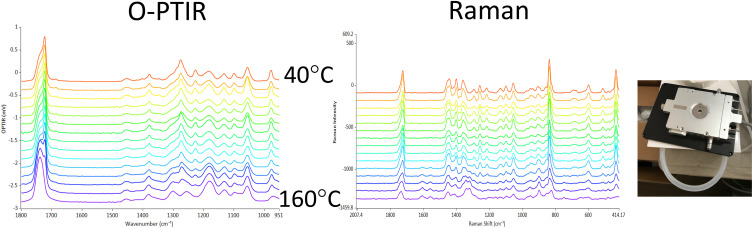
Submicron O-PTIR and simultaneous Raman spectra a biodegradable polymer under temperature ramp with an environmental stage (right). The spectra were acquired in reflection mode from 40 to 160 °C at 10 °C increments. The sample is courtesy of Isao Noda and data are courtesy of Photothermal Spectroscopy Corp.; reprinted with permission.

The measurement of samples under environmental perturbation(s), such as temperature (heating/cooling) or exposure to gases is a common and important experimental approach often applied to the study of polymers and catalysts. As is commonly employed in FTIR and Raman, environmental cells, capable of heating or cooling the sample and capable of introduction gases are also applicable directly with O-PTIR, with the added benefit of multi-modality with Raman and fluorescence imaging. The submicron simultaneous measurement of IR and Raman spectra while a polymer is heated to melting is shown in Fig. 35. This demonstrates the utility of employing complementary spectroscopic techniques such as IR and Raman to probe the structural changes a sample undergoes during such perturbations.

#### Battery research

**FIG. 36.**
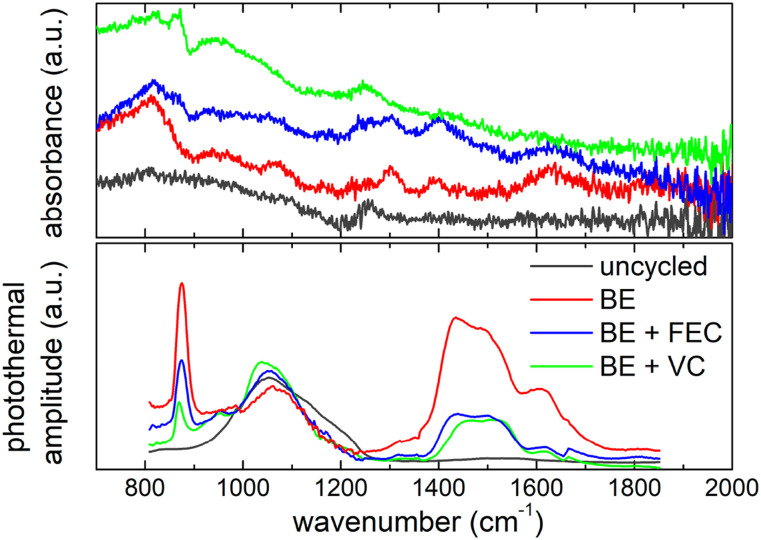
Comparison of ATR spectra (top) and O-PTIR spectra (bottom) on silicon nanowire electrodes, before and after electrochemical cycling with a benchmark electrolyte (BE) and the same electrolyte with two different additives vinylene carbonate (VC) and fluoroethylene carbonate (FEC). O-PTIR spectra revealed the production of carbonates and LiPF6 decomposition residues. The spectra are normalized at Si–C stretching vibration band (990 cm^−1^). Reprinted with permission from Sarra *et al.*, Batteries **9**(3), 148 (2023). Copyright 2023 Author(s), licensed under a Creative Commons Attribution 4.0 License.

O-PTIR has also been used in battery research, for example, studying spectroscopic changes in silicon nanowire electrodes under electrochemical cycling.^119^ Battery research represents a challenge for traditional infrared spectroscopy because many battery electrode materials are opaque to infrared radiation and thus cannot be studied in transmission. As such, techniques such as reflection or attenuated total reflection (ATR) must be used, which can lead to dispersive/scattering artifacts and/or lower SNR. Figure 36 shows a comparison of spectra acquired by conventional ATR infrared and O-PTIR on nanowire electrodes before and after cycling in the presence of different electrolyte additives. This first application of O-PTIR on lithium battery research demonstrated both better SNR and the ability to probe deeper into electrode materials compared to ATR measurements on the same sample. The improved SNR and improved depth sensitivity together enabled more complete analysis of reaction products than possible with conventional infrared approaches, demonstrating substantial benefits even when achieving higher spatial resolution is not the key focus.

#### Catalysis

**FIG. 37.**
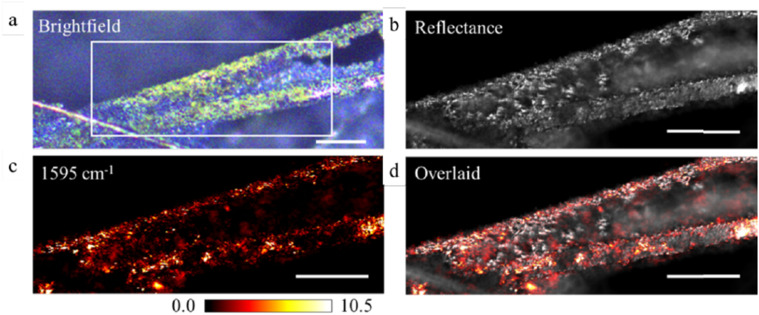
O-PTIR super-resolution IR imaging characterization of a copper–cobalt (CuCo) alloy electrocatalyst. (a) Brightfield image. (b) Reflectance image of the area indicated by the white box in panel (a) with 532 nm laser illumination. (c) Super-resolution IR imaging of the same area by O-PTIR imaging at 1595 cm^−1^ associated with the CuCo catalyst band. (d) Overlay of reflectance and IR image. Scale bars: 50 *µ*m. Reprinted with permission from Wu *et al.*, ACS Sustain. Chem. Eng. **10**(44), 14539 (2022). Copyright 2022 American Chemical Society.

O-PTIR measurements have also been used to study catalysts for waste water treatment for nitrate to ammonia conversion^120^ and dye degradation associated with printing and dyeing industries.^121^ Wu *et al.* used O-PTIR as part of a suite of analytical techniques, including x-ray diffraction, atomic force microscopy, electron microscopy, and conventional infrared spectroscopy, to evaluate copper–cobalt alloy electrocatalysts. O-PTIR was particularly used for morphological and compositional analysis of the catalyst material. Figure 37 shows O-PTIR chemical imaging of a copper–cobalt alloy electrocatalyst for ammonia conversion revealing the spatial localization of the active catalyst regions.

### Photonics/plasmonics

#### Perovksite/solar cells

**FIG. 38.**
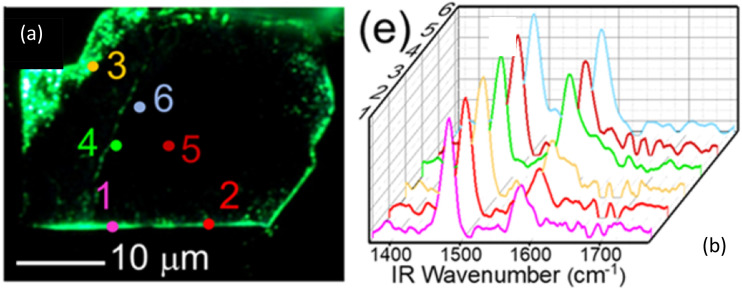
O-PTIR analysis of perovskite crystals for solar cell applications. O-PTIR spectra (b) were measured at locations identified in photoluminescence imaging (a). Reprinted with permission from Qin *et al.*, Chem. Mater. **32**(12), 5009 (2020). Copyright 2020 American Chemical Society.

O-PTIR has also been used to study perovskite materials, for example, to investigate the structures at the edges of a type of solar cell material known as 2D Ruddlesden–Popper perovskites^122^ (Fig. 38). This research discovered that at perovskite crystal edges, the material spontaneously transforms from a two-dimensional to a three-dimensional form. When combined with O-PTIR, it helped identify the loss of organic components (specifically butylammonium) and the presence of methylammonium in the 3D structures at the edges of perovskite crystals. This transformation is significant because it enhances the material’s ability to emit light and improves its electrical properties, which are crucial for increasing the efficiency of solar cells and other electronic devices. While the measurements shown in the following were performed on separate instruments, the current generation O-PTIR instruments optionally include fluorescence microscopy capabilities, which, with appropriate filter sets, can also perform photoluminescence imaging, thus enabling photoluminescence-guided infrared spectroscopy with sub-micron spatial resolution.

### Surface enhanced O-PTIR

**FIG. 39.**
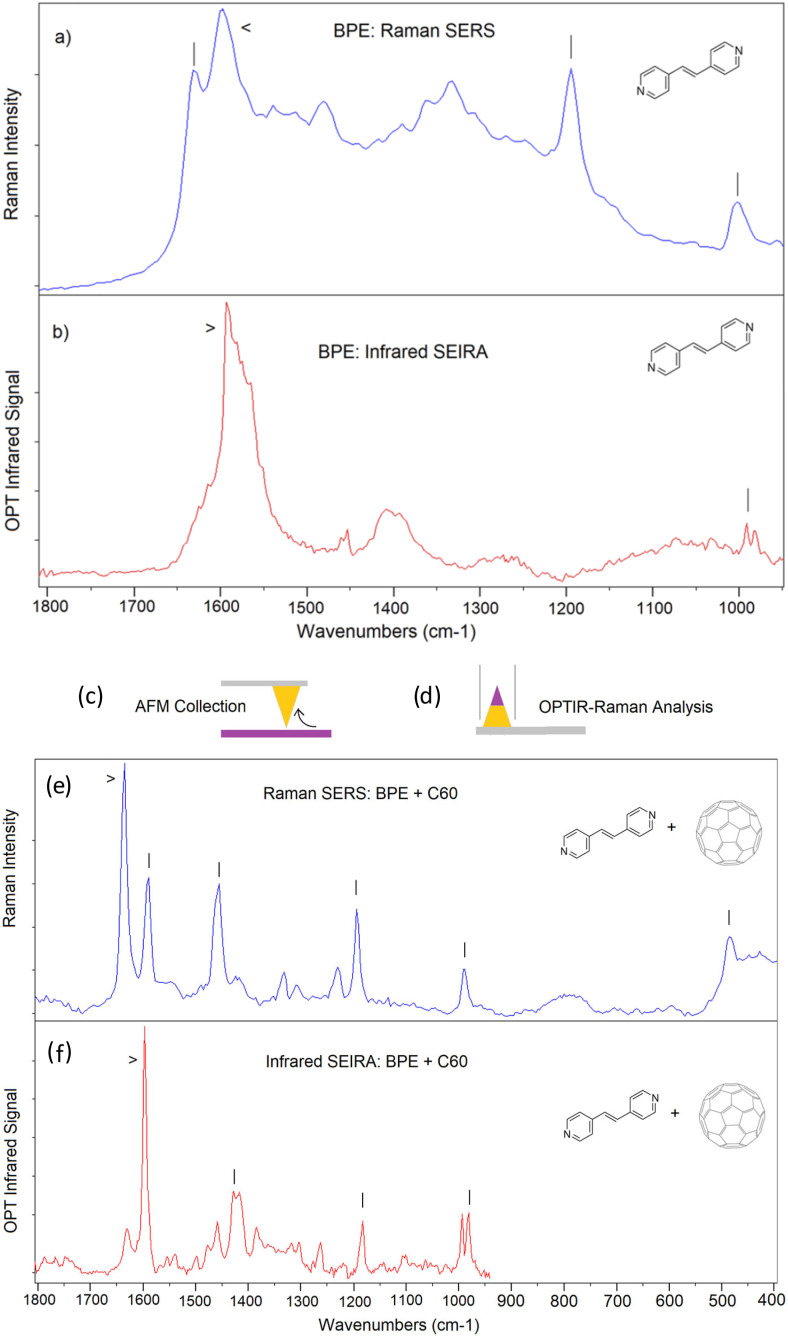
O-PTIR with plasmonic enhancement demonstrating single molecule sensitivity. Single molecular SERS (a) and SEIRA (b) spectra simultaneously acquired on 1,2-bis(4-pyridyl) ethylene (BPE) cast onto silver nanospheres. (c) AFM-based analyte nano-sampling and (d) plasmonic enhancement. SERS (e) and SEIRA (f) spectra on AFM nano-sampled/enhanced BP+C60 analytes. Reprinted with permission from Anderson, Rev. Sci. Instrum. **94**(2), 025103 (2023). Copyright (2023) Author(s), licensed under a Creative Commons Attribution 4.0 License.

Anderson recently demonstrated simultaneous and complementary surface-enhanced infrared absorption (SEIRA) and surface-enhanced Raman spectroscopy (SERS) using a Raman-enabled O-PTIR instrument in combination with plasmonically active substrates to achieve single molecule detection sensitivity.^123^ To demonstrate single molecule sensitivity, Anderson coated silver nanospheres with two target analytes [C60 and 1,2-bis(4-pyridyl) ethylene, BPE] and then measured them at sub-monolayer dilution to provide a high likelihood that observed hotspots contained only a single analyte molecule. As further evidence of single molecule sensitivity, Anderson also analyzed binary mixtures of C60 and BPE at low dilution, obtaining spectra that showed the spectral signatures of either C60 or BPE without contributions for the other analyte. Figures 39(a) and 39(b) show an example of concurrent single molecule SEIRA and SERS measurements on BPE. While single molecule SERS spectroscopy is well-established, to our knowledge, this is the first demonstration of single molecule detection by SEIRA and the first demonstration of single molecule spectroscopy by both Raman and infrared techniques. Since IR and Raman are highly complementary approaches, as described earlier, this demonstration is an extremely promising milestone. Anderson also showed how gold-coated atomic force microscopy probes could be used as both an analyte nano-sampler and as a plasmonic enhancer [Figs. 39(c)–39(f)] for O-PTIR based SEIRA and SERS measurements, albeit with reduced plasmonic enhancement compared to the silver nanospheres.

### Geochemical and paleontological applications

**FIG. 40.**
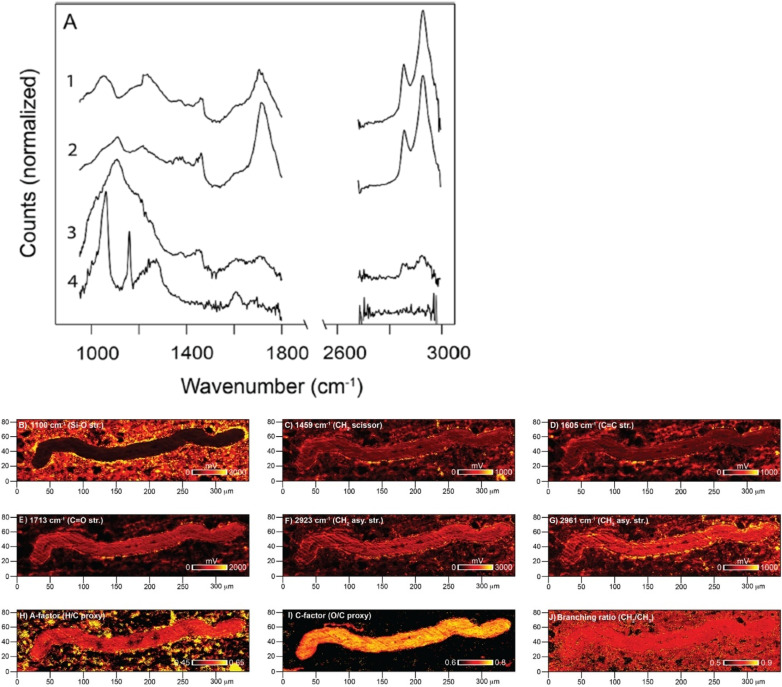
O-PTIR chemical characterization and visualization of organics in sediments associated with algal microfossils. (a) O-PTIR spectra on Tasmanites microfossil (spectra 1-2) and adjacent sediment regions (spectra 3-4). (b)–(j) O-PTIR absorption images and image ratios for relevant organic and inorganic absorption bands. Reprinted with permission from Jubb *et al.*, Org. Geochem. **177**, 104569 (2023). Copyright 2023 Author(s), licensed under a Creative Commons Attribution 4.0 License.

O-PTIR has also been used to enable the molecular characterization of sedimentary organic matter and organic fossils. Jubb *et al.*^124^ have used O-PTIR to map the distribution of functional groups in sedimentary organic matter (SOM) within the Upper Devonian Ohio Shale (Fig. 40), revealing that O-PTIR can discriminate between different types of organic materials and mineral matrices at sub-micron scales providing relevant insights into petroleum generation processes and sedimentary environments. In particular, the research mapped infrared functional groups associated algal microfossils and discriminated organic matter from adjacent solid bitumen with 500 nm spatial resolution.

### Failure analysis/defect identification

**FIG. 41.**
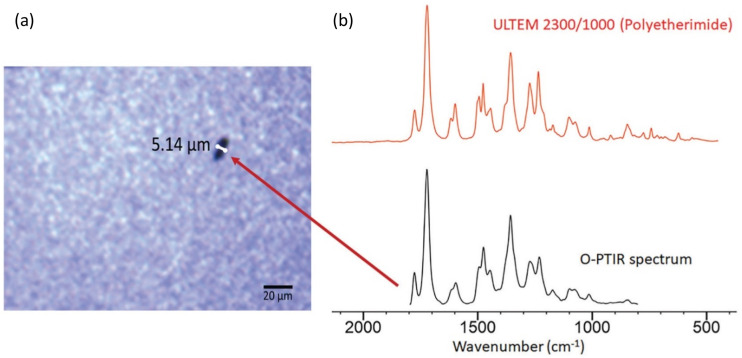
O-PTIR analysis of organic contaminant for data storage applications. (a) Optical image showing a ∼5 *µ*m defect on the surface of a disk drive recording head. (b) O-PTIR spectrum (bottom) and reference spectrum from KnowItAll spectral database, revealing an identification of polyetherimide. Reprinted with permission from Kansiz *et al.*, Microsc. Today **28**(3), 26 (2020). Copyright 2020 Microscopy Society of America.

**FIG. 42.**
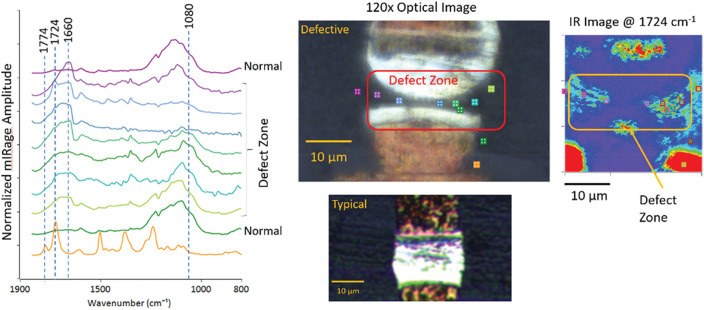
O-PTIR spectra and images associated with characterizing a defective weld in an electronic component. Optical images (center) show a good weld and a defective weld in an electronic component. O-PTIR spectra were collected at the color-coded locations in the image in and around the weld defect (left). The IR absorbance image (right) was collected at 1724 cm^−1^, where a properly formed weld has a strong absorption band. The spectra and images provide insights into the bond failure mechanism. Reprinted with permission from Kansiz *et al.*, Microsc. Today **28**(3), 26 (2020). Copyright 2020 Microscopy Society of America.

**FIG. 43.**
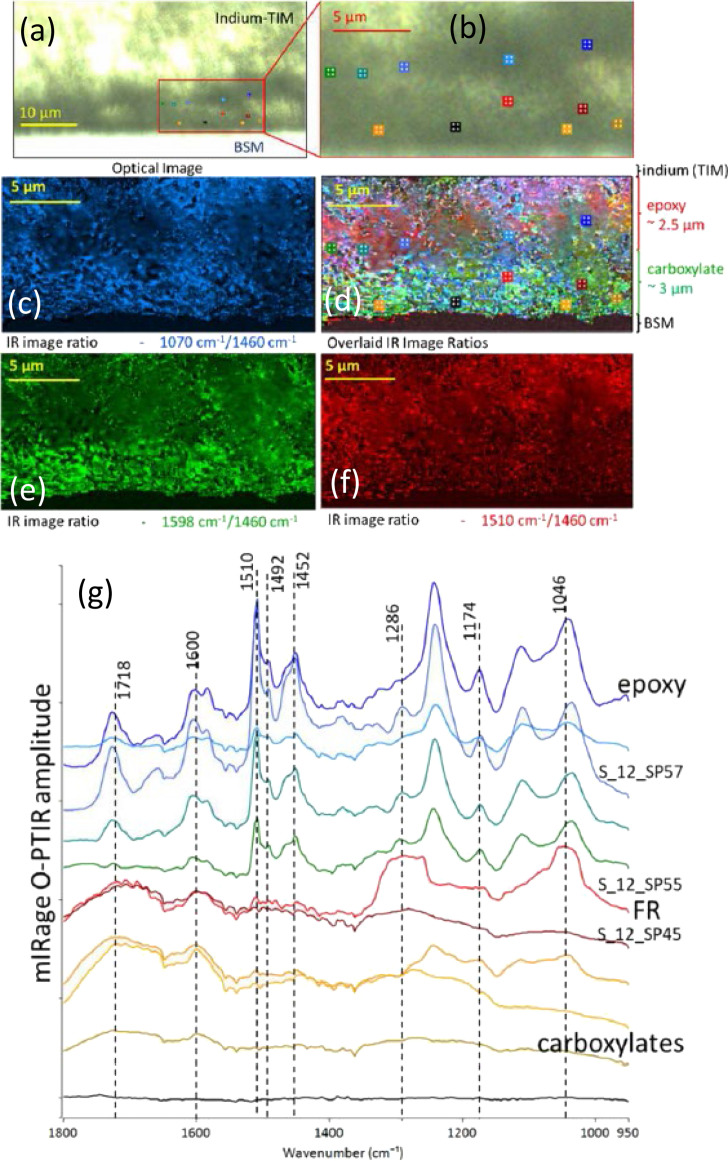
O-PTIR analysis of a cross-sectioned semiconductor device. (a) and (b) Optical image of the analyzed region of the cross sectioned device sample with locations noted for spectra in panel (g). (c), (e), and (f) O-PTIR ratiometric images at the wavenumbers indicated. (d) RGB overlay of ratio images in panels (c), (e), and (f) reprinted with permission from Zulkifli *et al.*, in *2022 IEEE International Symposium on the Physical and Failure Analysis of Integrated Circuits (IPFA)* (IEEE, 2022), p. 1. Copyright 2022 IEEE.

O-PTIR is being rapidly adopted in industrial research, including by Fortune 500 companies, often for process development, optimization, failure analysis, and defect identification.^125–129^ Applications include semiconductor,^129^ data storage,^24^ display, electronic, and pharmaceutical industries. A very common application is the identification of organic contaminants that are below the detection limit of conventional infrared spectroscopy. While electron microscopes configured with energy dispersive x-ray capabilities can routinely provide elemental analysis, there has been a gap in the ability to characterize and identify organic contaminants below the detection limit of conventional IR and Raman spectroscopy. O-PTIR is demonstrating significant success in this arena with its ability to characterize even sub-micron contaminants. Figure 41 shows an example of O-PTIR identification of a defect found on a disk drive recording head. Another common application is failure analysis associated with the degradation and/or migration of species within a device, and/or manufacturing related defects. Figure 42 shows an example of O-PTIR spectroscopy and chemical imaging associated with a defective weld in an electronic device. Figure 43 shows O-PTIR imaging and spectroscopic analysis of a packaged semiconductor device that has undergone underfill creep.

## ADVANCED MODALITIES AND OUTLOOK

O-PTIR researchers are continuing to push the state of the art and have demonstrated a variety of advanced modalities, including interferometric and phase-based detection and wide-field detection approaches^50,130–138^ as well as depth-resolved O-PTIR tomography.^58,139,140^ Looking ahead, we anticipate a panel of exciting directions of this technology. First, advanced infrared lasers and modalities are to be developed to further improve the sensitivity, speed, and enlarge the field of view of the technique. By sync-scan of IR pump and visible probe beam, video rate imaging speed has been achieved recently.^141^ With high-energy IR pulses, we expect OPTIR to reach millimeter-scale field of view for high-throughput tissue analysis. We expect infrared absorbing tags and substrates to be extensively developed for O-PTIR imaging of specific molecules and/or cellular processes without the need of click chemistry. We also expect the development of highly sensitive temperature reporters to boost the sensitivity of O-PTIR imaging toward a single molecule level. We expect the integration of OPTIR with microfluidics for high-throughput single cell or single particle analysis. Finally, we predict clinical translation as the next frontier through miniaturization of the O-PTIR microscope toward molecular-based diagnosis in the clinic settings.

## Data Availability

As this is a tutorial/review article, requests for source data should be directed to authors of cited publications.
